# DNA Barcoding and Morphometry Reveal Further Cryptic Bio-Diversity within the Pin Nematode Genus *Paratylenchus* (Nematoda: Tylenchulidae) †

**DOI:** 10.3390/plants11233385

**Published:** 2022-12-05

**Authors:** Juan Emilio Palomares-Rius, Antonio Archidona-Yuste, Ilenia Clavero-Camacho, José A. Carreira de la Fuente, Ana Rey, Benjamín Viñegla, Gracia Liébanas, Carolina Cantalapiedra-Navarrete, Pablo Castillo

**Affiliations:** 1 Institute for Sustainable Agriculture (IAS), Spanish National Research Council (CSIC), Avenida Menéndez Pidal s/n, Campus de Excelencia Internacional Agroalimentario, ceiA3, 14004 Córdoba, Spain; 2 Andalusian Institute of Agricultural and Fisheries Research and Training (IFAPA), Centro Alameda del Obispo, 14004 Córdoba, Spain; 3 Department of Animal Biology, Plant Biology and Ecology, University of Jaén, Campus ‘Las Lagunillas’ s/n, Edificio B3, 23071 Jaén, Spain; 4 Department of Biogeography and Global Change, Museo Nacional de Ciencias Naturales—CSIC, José Abascal 2, 28006 Madrid, Spain

**Keywords:** cytochrome c oxidase c subunit 1 (COI), cryptic species, D2-D3 expansion domains of the large ribosomal subunit (28S), internal transcribed spacer (ITS), new species

## Abstract

*Paratylenchus* species are obligate ectoparasitic nematodes on cultivated and wild herbaceous and woody plants occupying numerous soil categories. Several species may cause damage to several crops (*viz*. *P. dianthus*, *P. enigmaticus*, *P. microdorus*, *P. hamatus* and *P. epacris* on carnation, lettuce, rose and walnut, respectively). This investigation proves and emphasizes the relevance of applying integrative taxonomy for the accurate detection of *Paratylenchus* species in mountainous wild environments in the Malaga province, Southern Spain. This research analyzed 45 soil samples of maritimus pine and one of green heather in southern Spain and identified fourteen *Paratylenchus* species, two of them are described herein as new species (*P. paraaonli* sp. nov., *P. plesiostraeleni* sp. nov.), six of them were first reports for Spain (*P. canchicus*, *P. nainianus*, *P. neonanus*, *P. salubris*, *Paratylenchus* sp. 2 SAS, and *P. wuae*), and six species (*P. caravaquenus*, *P. microdorus*, *P. nanus*, *P. neoamblycephalus*, *P. sheri*, and *P. variabilis*) have been already reported in Spain. Accordingly, these data increase the biodiversity of pin nematodes in Spain comprising a total of 47 species (33.1% out of 142 total species of this genus). Phylogenetic analyses based on ribosomal and mitochondrial markers (D2-D3, ITS, and partial COI) resulted in a consistent position for the newly described *Paratylenchus* species in this study (*P. plesiostraeleni* sp. nov., *P. paraaonli* sp. nov.). *Paratylenchus plesiostraeleni* sp. nov. grouped in a separated subclade as unequivocal species from the *P. straeleni*-complex species (including *P. straeleni* and *P. parastraeleni*), and *P. paraaonli* sp. nov. clustered with *P. vitecus*, but clearly separate from this species. This study indicates that *Paratylenchus* species diversity in natural environments may be higher than expected, and this study may help in accurate identifications.

## 1. Introduction

Pin nematodes (*Paratylenchus* Micoletzky, 1922) [1] comprises the largest genus within the family Tylenchulidae with more than 140 species, some of which are known to be significant agricultural plant parasites [2,3,4,5,6,7,8]. *Paratylenchus* species are root-ectoparasitic obligate nematodes of short body length (≤600 μm) with stylet length from 10 to 120 μm, and widely present in different natural habitats and crops, and worldwide distributed [2,3,4,5].

The biodiversity within *Paratylenchus* to date is insufficiently known, resulting in difficulties in identifications and incomplete inventories of species, particularly in wild environments [4,5,8]. Cryptic species within *Paratylenchus* have emerging biological evidence that is proposed for those species which disclose low morphological, but substantial genetic difference [3,4,5,9,10]. The separation of independent lineages within *Paratylenchus* is critical for taxonomy and species identification, but also for understanding the processes leading to the extensive diversity in the tree life [3,4,5,9,11]. Recent studies have demonstrated that integrative taxonomical approaches provide unequivocal molecular markers (fragments of nuclear ribosomal and mitochondrial DNA gene sequences) for the identification of different *Paratylenchus* cryptic species associated with a specific and common morphology and morphometry [3,4,5,9]. The species delimitation in this genus is a very difficult task because of the high morphological and morphometric similarity and the large number of species. Thus, the molecular data are needed in order to separate closely related morphometrically–morphologically species [8]. A prominent case of outstanding cryptic species diversity within *Paratylenchus* is the *P. straeleni-*complex species distinguishing 4–9 presumed species [3,4,5,9], including one new species recently described from southern Spain, *viz*. *P. parastraeleni* [5]. As pointed out in previous studies, the number of cryptic species within *Paratylenchus* is likely to be increased in forthcoming years, particularly, with studies focused on wild environments along with the increasing use of molecular markers for species identification [4,5].

In our previous studies on the biodiversity of the genus *Paratylenchus* in Spain, thirty-nine species have been reported mainly from cultivated fruit-trees including almond, apricot, cherry, nectarine and peach, and some natural ecosystems: *P. amundseni*, *P. aciculus*, *P. aonli*, *P. arculatus*, *P. baldaccii*, *P. caravaquenus*, *P. ciccaronei*, *P. enatus*, *P. enigmaticus*, *P. goodeyi*, *P. hamatus*, *P. holdemani*, *P. indalus*, *P. israelensis*, *P. macrodorus*, *P. microdorus*, *P. minusculus*, *P. mirus*, *P. nanus*, *P. neoamblycephalus*, *P. pandatus*, *P. parastraeleni*, *P. pedrami*, *P. peraticus*, *P. projectus*, *P. recisus*, *P. sheri*, *P. similis*, *P. steineri*, *P. straeleni*, *P. tateae*, *P. tenuicaudatus*, *P. teres*, *P. vandenbrandei*, *P. variabilis*, *P. veruculatus*, *P. verus*, *P. vitecus*, and *P. zurgenerus* [4,5].

This study is the third in a succession disentangling the cryptic diversity of pin nematodes in Spain with the final objective of unraveling the reliable biodiversity of these nematodes in wild areas in Southern Spain [4,5]. The current distribution of *Paratylenchus* in Spain, to about 90% of species only described in Southern Spain (35 out of 39 species, and 24 of them established by integrative taxonomy) indicates that this part of the country may be contemplated as a likely hotspot of biodiversity for *Paratylenchus* species [4,5].

The major aims of this research were to (i) precisely recognize the morphological and morphometrical methods for the new *Paratylenchus* populations found in a widespread nematode study on maritimus pine (*Pinus pinaster* Ait.) mountainous forests at the Malaga province (Southern Spain); (ii) provide molecular characterization of the detected *Paratylenchus* populations by means of ribosomal markers (D2-D3 expansion segments of 28S rRNA, Internal Transcribed Spacer region (ITS) rRNA) and the mitochondrial cytochrome c oxidase subunit 1 (COI); and (iii) investigate phylogenetic relationships within *Paratylenchus* spp. using ribosomal and mitochondrial molecular markers.

## 2. Results

Fourteen species were recognized from 27 populations of *Paratylenchus* spp. in 45 soil samples from the rhizosphere of maritimus pine (*Pinus pinaster* Ait.) mountainous forests and one sample from green heather (*Erica scoparia* L.) on three mountains in the Malaga province, Southern Spain (Table 1). Nematode populations and *Paratylenchus* species within each mountain were distributed as follows: the Bermeja-Crestellina Mountain (twelve populations, ten *Paratylenchus* species), Nieves Mountain (six populations, four *Paratylenchus* species), and Tejeda-Almijara Mountain (nine populations, seven *Paratylenchus* species) (Table 1). In these populations, all available life stages (females, males, and juveniles) were precisely characterized morphologically and morphometrically, together with molecular markers for their accurate identification (Table 1). Of the 27 populations of *Paratylenchus* spp., 5 populations were contemplated as new undescribed species and 22 were already described species (Table 1). The new species populations comprise two populations that are *Paratylenchus paraaonli* sp. nov. and three populations inside the *P. straeleni*-complex that were designated here as *Paratylenchus plesiostraeleni* sp. nov. The described species comprised *P. canchicus* Mohilal and Dhanachand, 2004, *P. caravaquenus* Clavero-Camacho et al., 2021, *P. microdorus* Andrássy, 1959, *P. nainianus* Edward & Misra, 1963, *P. neoamblycephalus* Geraert, 1965, *P. neonanus* Mathur et al., 1967, *P. salubris* Raski, 1975, *P. sheri* (Raski, 1973) Siddiqi, 1986, *Paratylenchus* sp. 2 SAS, *P. variabilis* Raski, 1975, and *P. wuae* Yu et al., 2016. Within these species, six need to be contemplated as first reports in Spain (namely *P. canchicus*, *P. nainianus*, *P. neonanus*, *P. salubris*, *Paratylenchus* sp. 2 SAS, and *P. wuae*) and measurements from females, and juveniles (if existing), as well as molecular markers were presented for their unambiguous diagnostics (Table 1).

### 2.1. Taxonomy

#### 2.1.1. Description of *Paratylenchus paraaonli* sp. nov.

**Figure 1:**
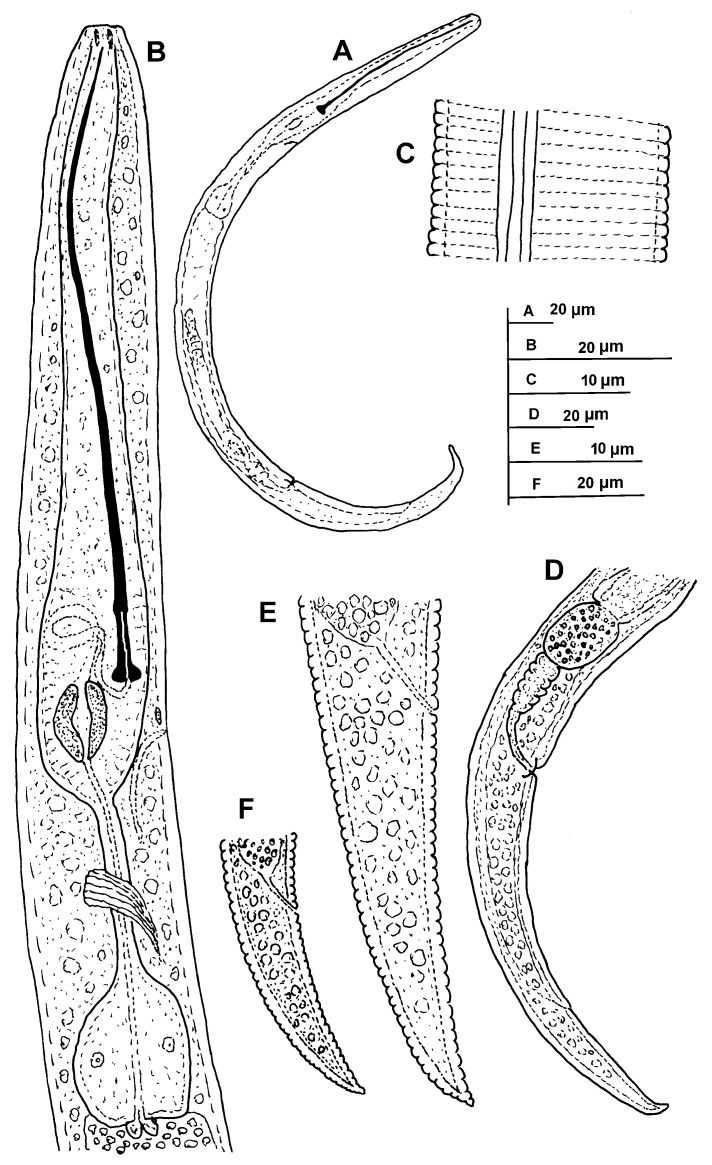
Line illustrations of *Paratylenchus paraaonli* sp. nov. (**A**) Whole female body; (**B**) Female pharyngeal region; (**C**) Detail of lateral field at mid-body; (**D**) Female posterior region; (**E**,**F**) Female tail.

**Figure 2:**
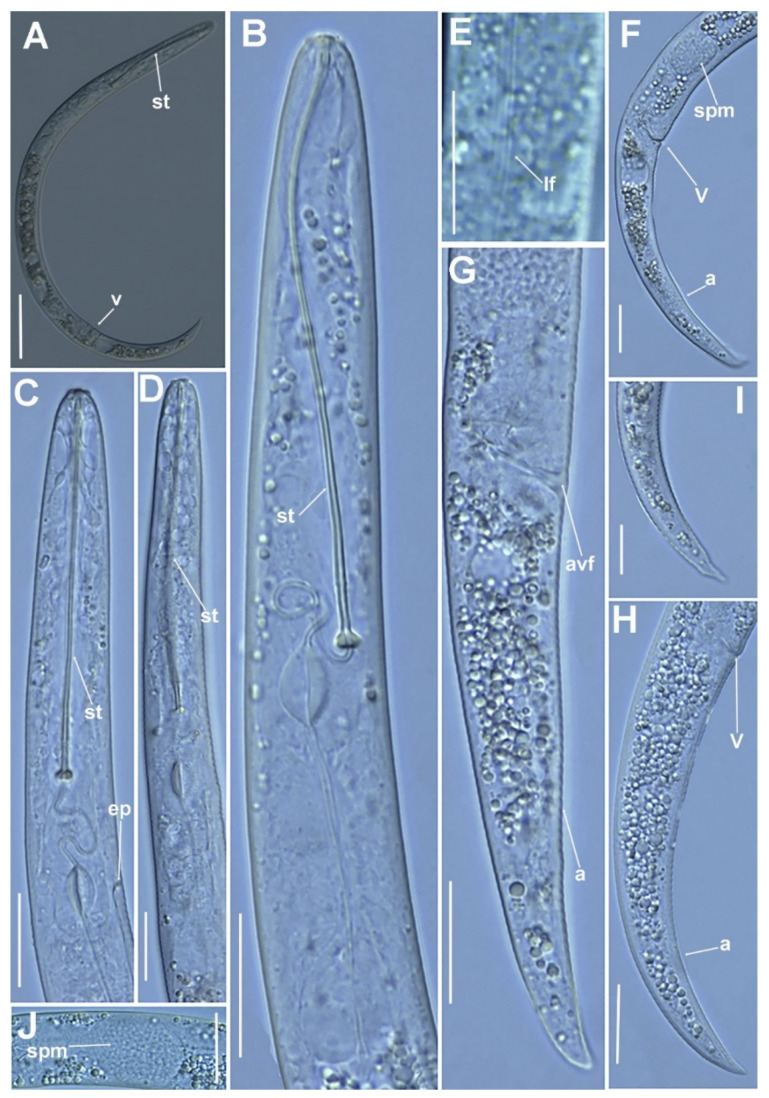
Micro-photomicrographs of *Paratylenchus paraaonli* sp. nov. female. (**A**) Whole female with stylet and vulva arrowed; (**B**) pharyngeal region; (**C**,**D**) detail of female stylet region; (**E**) detail of lateral fields; (**F**–**H**) female posterior region with vulva and anus (arrowed) and detail of vulva showing advulval flap (arrowed); (**I**) female tail; (**J**) detail of spermatheca (arrowed). Scale bars (**A** = 50 µm; **B**–**J** = 20 µm). (Abbreviations: a = anus; avf = advulval flap; ep = excretory pore; lf = lateral field; st = stylet; spm = spermatheca; st = stylet; V = vulva).

**Figure 3:**
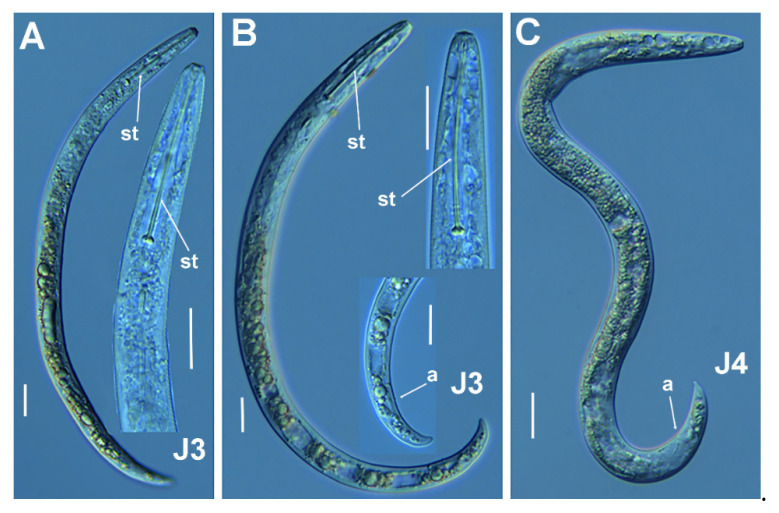
Micro-photomicrographs of *Paratylenchus paraaonli* sp. nov. third- and fourth-stage juveniles. (**A**,**B**) Entire third-stage juveniles showing stylet (arrowed), and insert of pharyngeal region and tail; (**C**) Entire fourth-stage juvenile showing pharyngeal region without stylet. Scale bars (**A**–**C** = 20 µm). (Abbreviations: a = anus; st = stylet).

(Figure 1, Figure 2 and Figure 3, Table 2). http://zoobank.org/urn:lsid:zoobank.org:act:79A40E43-1D8C-44FA-83F8-84A92418F3D8 (accessed on 21 September 2022).

*Female*: body delicate, body habitus after heat relaxation ventrally arcuate to assemble an open C; cuticle softly annulated; lateral field with four distinct smooth lines equidistantly separated and forming three bands. Lip region continuous with the rest of the body, conoid-truncate, with submedian lobes small; and very slight sclerotization. Stylet long, delicate and flexible, 18.2–24.8% of body length, conus 5.7–10.5 times longer than shaft, 85.1–91.3% of total stylet length. Stylet knobs rounded, laterally directed, small, 3.0–3.5 μm across. Pharynx well developed, procorpus cylindrical, 60–70 μm long. Secretory excretory pore situated at level of the large sclerotized valve. Hemizonid visible, placed one to two annuli anterior to excretory pore. Pharyngeal valves 9.0–10.0 μm long, located at 61.8–74.8% of pharynx length from anterior end. Basal bulb pyriform, 9.0–11.0 μm wide, 14.0–16.0 μm high. Ovary outstretched, spermatheca elongate-oval, 17 (13–25) μm long, 11 (9–17) μm wide, occupied with 1.0–1.5 μm in diameter round sperm. Advulval flap membranes small, 3.5–4.0 μm long. Elongate-conoid tail with finely to broadly round terminus, about half vulva–anus distance (0.4–0.6).

*Male*: not found, but the spermatheca was detected filled with sperm in several specimens, suggesting that males are essential for reproduction but were not detected in this survey.

*Juveniles*: J3 and J4 were detected with similar morphology to adult females (Figure 3). J3 bearing flexible stylet 46.3 (43.0–49.0) μm-long, and a functional pharynx, well developed. However, in J4, stylet is absent, and pharynx is not functional with numerous granular body content (Figure 3), representing the resting stage.

**Table 2:** Morphometrics of *Paratylenchus paraaonli* sp. nov. paratype females, third- and fourth-stage juveniles, and an additional population. All measurements are in µm and in the form: mean ± s.d. (range).

	Holotype		Paratypes		
	Female	Females	Juveniles (J3)	Juveniles (J4)	Females
Sample code	WPPp4	WPPp4	WPPp4	WPPp4	WPPp3
Locality	Casares, Malaga				Casares, Malaga
n	1	18	4	2	3
L	345	340.4 ± 27.8 (278–380)	315.8 ± 12.7 (300–331)	(340, 367)	347.7 ± 19.2 (327–365)
a *	21.6	21.5 ± 1.4 (18.5–24.3)	20.3 ± 1.3 (18.4–21.4)	(19.4, 20.4)	22.0 ± 2.3 (19.8–24.3)
b	2.6	2.6 ± 0.1 (1.4–3.0)	2.9 ± 0.1 (2.8–3.1)	(4.3, 4.5)	2.7 ± 0.2 (2.5–3.0)
c	11.5	12.1 ± 1.2 (10.4–14.0)	14.5 ± 0.4 (14.1–15.0)	(18.9, 22.9)	13.1 ± 1.6 (11.3–14.0)
c’	3.5	3.5 ± 0.4 (3.1–4.4)	2.6 ± 0.2 (2.4–2.9)	(2.0, 2.1)	3.4 ± 0.2 (3.3–3.6)
V	77.4	75.7 ± 1.2 (72.8–77.4)	-	-	75.8 ± 1.1 (74.5–76.8)
G1	33.3	31.7 ± 2.6 (27.0–37.1)	-	-	31.2 ± 1.2 (29.9–32.1)
Stylet length	73.0	72.2 ± 3.1 (67.0–79.0)	46.3 ± 2.8 (43.0–49.0)	-	72.0 ± 1.7 (71.0–74.0)
(Stylet length/body length) × 100	21.2	21.3 ± 1.5 (18.2–24.8)	14.7 ± 0.8 (13.6–15.6)	-	20.7 ± 1.2 (19.5–21.7)
Conus length	65.0	64.1 ± 3.2 (57.0–69.0)	39.5 ± 2.4 (37.0–42.0)	-	62.7 ± 1.1 (62.0–64.0)
m	89.0	88.7 ± 1.6 (85.1–91.3)	85.4 ± 0.7 (84.4–86.0)	-	87.0 ± 0.5 (86.5–87.3)
DGO	7.0	8.1 ± 0.72 (7.0–9.5)	5.3 ± 0.3 (5.0–5.5)	-	7.2 ± 0.3 (7.0–7.5)
O	9.6	11.2 ± 1.0 (9.5–12.7)	11.4 ± 0.2 (11.1–11.6)	-	10.0 ± 0.6 (9.5–10.6)
Lip width	4.5	4.6 ± 0.2 (4.5–5.0)	4.3 ± 0.3 (4.0–4.5)	(4.5, 5.0)	4.5 ± 0.0 (4.5–4.5)
Median bulb length	23.0	24.2 ± 2.1 (22.0–29.0)	-	-	23.3 ± 0.6 (23.0–24.0)
Median bulb width	11.0	11.0 ± 1.3 (9.0–14.0)	-	-	11.3 ± 0.6 (11.0–12.0)
Anterior end to center median bulb	98	88.5 ± 6.3 (77.0–100.0)	-	-	85.0 ± 3.0 (82.0–88.0)
MB	74.8	67.5 ± 3.2 (61.8–74.8)	-	-	66.4 ± 0.4 (65.9–66.7)
Nerve ring to anterior end	111.0	108.2 ± 6.5 (97.0–120.0)	-	-	103.7 ± 5.9 (97.0–108.0)
Excretory pore to anterior end	93.0	88.5 ± 6.2 (79.0–103.0)	86.8 ± 9.3 (77.0–99.0)	(74.0, 76.0)	88.0 ± 3.5 (86.0–92.0)
Pharynx length	131.0	130.7 ± 7.7 (115.0–145.0)	108.5 ± 6.5 (103.0–117.0)	(79.0, 81.5)	128.0 ± 4.6 (123.0–132.0)
Maximum body diam.	16.0	15.9 ± 1.4 (14.0–19.0)	15.6 ± 1.7 (14.0–18.0)	(17.5, 18.0)	15.8 ± 0.8 (15.0–16.5)
Vulva–anus distance	58	57.8 ± 6.0 (46.0–66.0)	-	-	58.7 ± 1.5 (57.0–60.0)
Tail length	30.0	28.4 ± 3.5 (22.0–35.0)	21.8 ± 0.6 (21.0–22.5)	(16.0, 18.0)	26.7 ± 2.1 (25.0–29.0)
Anal body diam.	8.5	8.2 ± 0.9 (7.0–10.0)	8.4 ± 0.8 (7.5–9.0)	(8.0, 8.5)	7.8 ± 0.3 (7.5–8.0)

* Abbreviations: a = body length/greatest body diameter; b = body length/distance from anterior end to pharyngo-intestinal junction; DGO = distance between stylet base and orifice of dorsal pharyngeal gland; c = body length/tail length; c’ = tail length/tail diameter at anus or cloaca; G1 = anterior genital branch length expressed as percentage (%) of the body length; L = overall body length; m = length of conus as percentage of total stylet length; MB = distance between anterior end of body and center of median pharyngeal bulb expressed as percentage (%) of the pharynx length; n = number of specimens on which measurements are based; O = DGO as percentage of stylet length; V = distance from body anterior end to vulva expressed as percentage (%) of the body length.

Diagnosis and Relationships

*Paratylenchus paraaonli* sp. nov. can be delineated by lateral field with four lines, withadvulval flap membranes, and a discreetly long and flexible female stylet of 72.2 (67.0–79.0) µm. Lip region continuous with the rest of the body, conoid-truncate, with submedian lobes small; with scanty sclerotization. Spermatheca elongated, oval. Elongate-conoid tail with finely to broadly rounded terminus. It belongs to group 10 by Ghaderi et al. [2], characterized by a long stylet (>40 µm), four lateral lines and present advulval flaps.

The morphology and morphometry, *P. paraaonli* sp. nov. is almost identical to *P. aonli* and is also similar to *P. brasiliensis* and *P. marylandicus*. Although general morphology and many matrix codes of *P. paraaonli* sp. nov. by the polytomous key by Palomares-Rius et al. [8] are quite similar to *P. aonli*, both species can be separated by the length of stylet (67.0–79.0 µm vs. 55.0–65.0 µm), excretory pore location (at pharyngeal valve bulb vs. at isthmus level), V ratio (72.8–77.4 vs. 76–84), vulva anus distance (46.0–66.0 µm vs. 26 µm), tail length (22.0–35.0 µm vs. 21 µm), and c ratio (10.4–14.0 vs. 14–20) [12]. Interestingly, *P. aonli* has been already reported in Navarra, northern Spain, and could be of interest to confirm this identification by integrative taxonomical approaches [13]. It differs from *P. brasiliensis* by body length (278–380 µm vs. 220–250 µm), length of stylet (67.0–79.0 µm vs. 58.0–62.0 µm), tail length (22.0–35.0 µm vs. 18 µm), and tail shape (elongate-conoid with finely to broadly rounded terminus vs. conoid, terminus sharply pointed, with clear area of variable size) [14]. It differs from *P. marylandicus* by stylet length (67.0–79.0 µm vs. 63.0–71.0 µm), excretory pore position (at pharyngeal valve bulb vs. at isthmus level), body diameter at post-vulval region (normal vs. marked reduction in post-vulval body diameter), tail length (22.0–35.0 µm vs. 40 µm), and c ratio (10.4–14.0 vs. 9–12) [15]. According to the polytomous key of Palomares-Rius et al. [8], matrix codes for the new species are (codes in parentheses are exceptions): A3(4), B1, C3, D1, E4, F2, G2, H2, I2(3), J2, K?, L?, M3(4,5), N1(2), O3(2,4), P?, Q2, R1(2), S2(1), T1, U1(2), V1, W1, X1(2).

Molecular Characterization

Eight D2-D3 of the 28S rRNA (ON873196-ON873203), five ITS (ON873174-ON873178), and nine COI gene sequences (ON873174-ON873182) were sequenced for these new taxa. Overall intraspecific variation was 7 to 9 nucleotides (99.0–98.7% similarity) for D2-D3, a 99.7–100.0% similarity (0–2 nucleotides and 0 indels) for ITS, and a 98.9–100.0% (0–4 nucleotides, 0 indels) for COI. The closest species to *P. paraaonli* sp. Nov. were *P. vitecus*, being 96.1–96.3% similar for the D2-D3 region from Spain (MZ265136-MZ265141) (differing 26–27 nucleotides and 4 indels) [5], followed with a 95.6% similarity to *P. teres* from Iran (MN088376) (differing 33 nucleotides and 5 indels) [16], and with a 93.2–90.8% similarity (differing 62–66 nucleotides and 2–4 indels) to *P. wuae* from Canada and China (KM061782, MW041155) [17]. ITS region was 90.1% similar to *P. vitecus* from Spain (MZ265059-MZ265062), 88.9% similar to *P. pandatus* (MZ265041-MZ265042), 87.3% similar to *P. macrodorus* (MZ265034-MZ265038), and 87.3% similar to *P. wuae* (KM061783) (differing in 58 to 86 nucleotides, 13 to 20 indels) [5,17]. For COI gene sequences, the similarity values were 90.2% (differing from 38 nucleotides and 0 indels) from *P. wuae* (MF770965), 90.1% (differing 34–35 nucleotides and 0–1 indel) from *Paratylenchus* sp. Ge16 PRS-2020 (MW421703-MW421704) and 92.0% (differing 27 nucleotides and 0 indel) from *P*. *pandatus* (MZ262247) [5,18]. All molecular markers studied clearly separate the new species from other *Paratylenchus* species. Unfortunately, no molecular data for *P. aonli* was provided in The National Center for Biotechnology Information (NCBI).

Type Habitat and Locality

*Paratylenchus paraaonli* sp. nov. was detected in the rhizosphere of maritimus pine (*Pinus pinaster* Ait.), coordinates 36°28’55.1″ N, 5°4′37.1″ W; in the municipal district of Casares, Malaga province, on the Bermeja-Crestellina Mountain, southeastern Spain. An additional sample from the same host plant and locality are stated in Table 1.

Etymology

The species name, *paraaonli*, refers to Gr. prep. para, alongside of and resembling, N.L. masc. n. *aonli*, since it is very close to *Paratylenchus aonli*.

Type Material

Female holotype, 14 female paratypes, 4 third-stage juveniles and 2 fourth-stage juveniles paratypes (slide numbers WPPp4-01, WPPp4-02 WPPp4-10) were deposited in the Nematode Collection of the Institute for Sustainable Agriculture, CSIC, Córdoba, Spain; two females at Istituto per la Protezione delle Piante (IPP) of Consiglio Nazionale delle Ricerche (C.N.R.), Sezione di Bari, Bari, Italy (WPPp4-11); and two females deposited at the USDA Nematode Collection (slide T-7736t).

#### 2.1.2. Description of *Paratylenchus plesiostraeleni* sp. nov.

**Figure 4:**
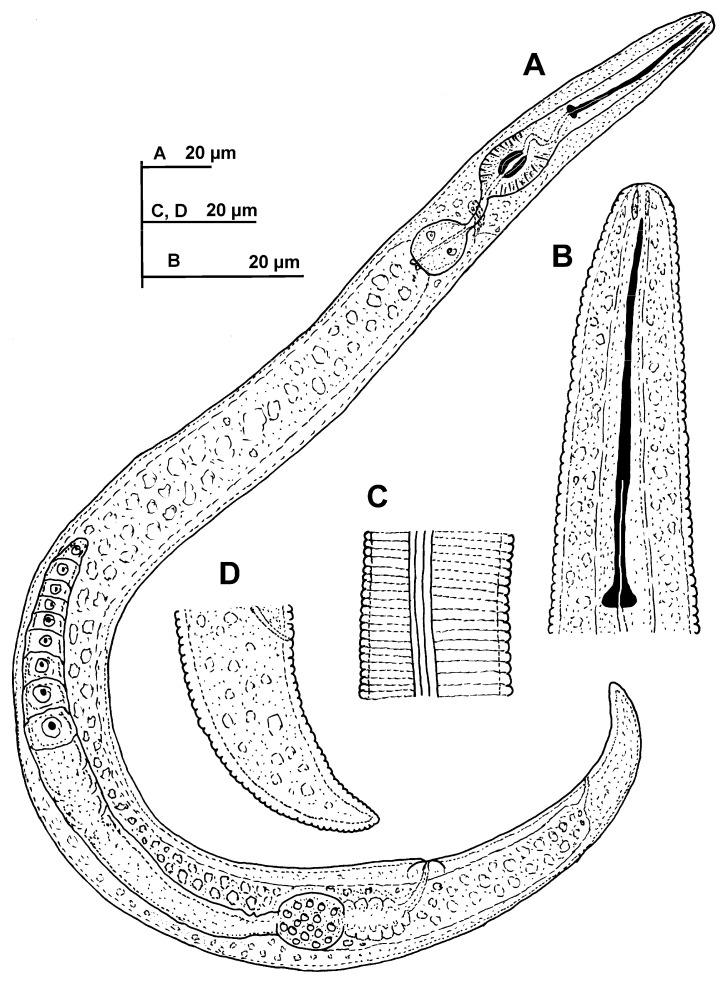
Line illustrations of *Paratylenchus plesiostraeleni* sp. nov. (**A**) Whole female; (**B**) Female stylet region; (**C**) Detail of lateral fields at mid-body; (**D**) Female tail.

**Figure 5:**
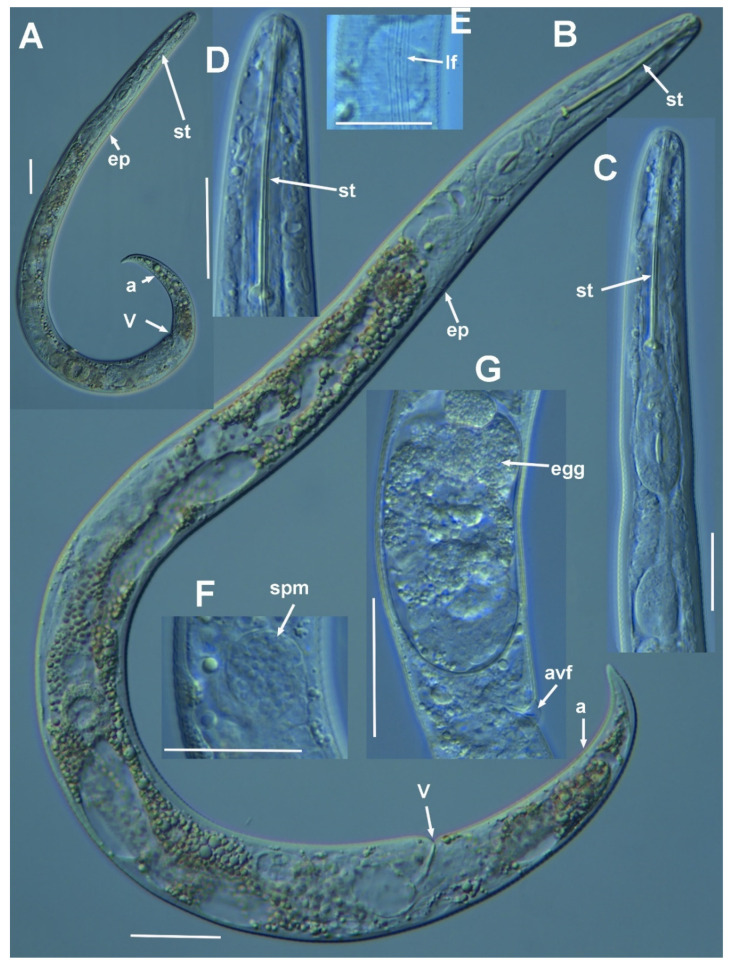
Micro-photomicrographs of *Paratylenchus plesiostraeleni* sp. nov. female. (**A**,**B**) Whole female with stylet, excretory pore and vulva arrowed; (**C**) pharyngeal region; (**D**) Stylet region; (**E**) Detail of lateral fields; (**F**) Detail of spermatheca; (**G**) Detail of vulva showing advulval flap (arrowed) and egg (arrowed). Scale bars (**A**–**G** = 20 µm). (Abbreviations: a = anus; avf = advulval flap; egg = egg; ep = excretory pore; lf = lateral field; spm = spermatheca; st = stylet; V = vulva).

**Figure 6:**
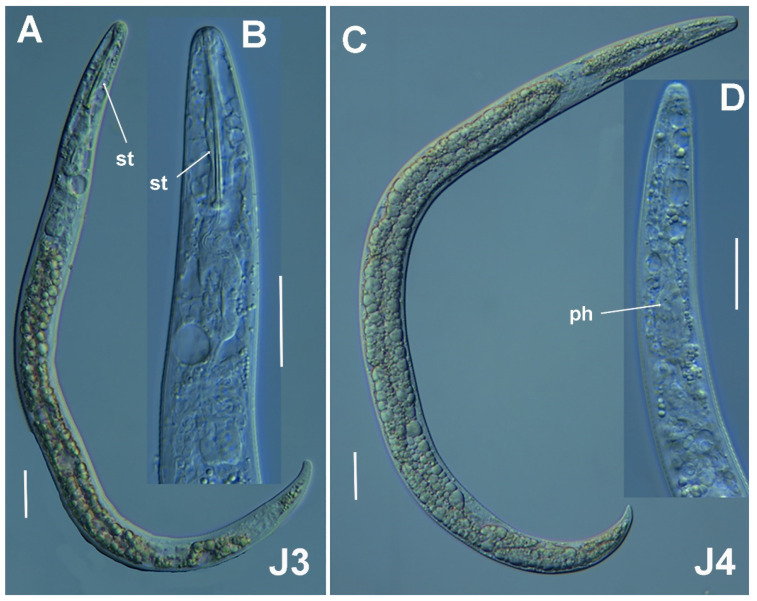
Micro-photomicrographs of *Paratylenchus plesiostraeleni* sp. nov. third- and fourth-stage juveniles. (**A**) Whole third-stage juvenile showing stylet (arrowed); (**B**) Pharyngeal region showing stylet (arrowed); (**C**) Whole fourth-stage juvenile showing absence of stylet and few developed pharynxes (arrowed). (**D**) Pharyngeal region of fourth-stage juvenile showing non-functional pharynx. Scale bars (**A**–**D** = 20 µm). (Abbreviations: ph = pharynx; st = stylet).

(Figure 4, Figure 5 and Figure 6, Table 3). http://zoobank.org/urn:lsid:zoobank.org:act:9B208E13-B4B9-4C6D-A56A-3C66BFCF5881 (accessed on 21 June 2022).

*Female*: body delicate, body habitus after heat relaxation ventrally arcuate to assemble a close C; cuticle softly annulated; lateral field with four distinct smooth lines equidistantly separated and forming three bands, 3.0–3.5 µm wide. Lip region continuous with the rest of the body, conoid-rounded, with submedian lobes almost indistinct; and very slight sclerotization. Stylet thin, flexible, covering 9.1–11.4% of body length, conus 2.5–2.9 times longer than shaft, 71.3–72.6% of entire stylet. Stylet knobs rounded, laterally directed, small, 3.0–3.5 μm across. Pharynx well developed, procorpus cylindrical, 50–58 μm long. Secretory excretory pore located at distal end of basal bulb. Hemizonid visible, placed one to two annuli forward to excretory pore. Pharyngeal valves 7.0–9.0 μm long, located at 60.5–74.0% of pharynx length from anterior end. Basal bulb pyriform, 12–13 × 16–17 µm long. Ovary outstretched, almost spherical, 13.8 (13.5–14.0) µm wide, occupied with 1.0–1.5 µm width rounded sperm. Advulval flap membranes well developed, 5.0–6.0 μm long. Conoid tail progressively narrowing to form a terminus subacute to finely rounded, corresponding to 0.5–0.8 times as long as the vulva–anus distance.

*Male*: not found, but sperm was detected filling the spermatheca in several specimens, suggesting that males are essential for reproduction but were not detected in this survey.

*Juveniles*: J3 and J4 were detected with similar body morphology to adult females (Figure 6). J3 bearing flexible stylet 37.7 (36.0–39.0) μm long, and pharynx well developed, functional. However, in J4, stylet is absent, and pharynx is not functional with a granular body content (Figure 6), representing the resting stage.

**Table 3:** Morphometrics of *Paratylenchus plesiostraeleni* sp. nov. paratype females, third- and fourth-stage juveniles, and additional populations. All measurements are in µm and in the form: mean ± s.d. (range).

	Holotype	Paratypes				
	Female	Females	Juveniles (J3)	Juveniles (J4)	Females	Females
Sample Code	CMPp4	CMPp4	CMPp4	CMPp4	WPPp4	EMPp6
Locality	Tolox, Malaga				Casares, Malaga	Canillas, Málaga
n	1	20	3	3	4	3
L	434	440.9 ± 45.8 (381–536)	310.3 ± 16.3 (299–329)	438.0 ± 70.1 (363–502)	412.8 ± 31.7 (384–458)	408.0 ± 42.5 (365–450)
a*	21.7	20.3 ± 2.4 (15.0–23.3)	15.5 ± 0.3 (15.2–15.7)	20.5 ± 0.4 (20.2–20.9)	20.7 ± 1.7 (18.8–22.4)	19.2 ± 3.2 (15.5–21.5)
b	3.7	4.0 ± 0.4 (3.4–4.4)	3.4 ± 0.1 (3.3–3.5)	4.6 ± 0.8 (3.7–5.2)	4.0 ± 0.3 (3.5–4.2)	3.6 ± 0.5 (3.2–4.1)
c	12.8	12.4 ± 1.9 (10.0–15.9)	10.3 ± 0.3 (10.1–10.6)	12.3 ± 1.2 (11.2–13.6)	13.3 ± 1.1 (12.4–14.8)	13.6 ± 0.8 (13.0–14.5)
c’	2.6	2.8 ± 0.3 (2.4–3.3)	2.6 ± 0.1 (2.5–2.6)	2.6 ± 0.2 (2.5–2.8)	2.7 ± 0.4 (2.4–3.2)	2.8 ± 0.3 (2.6–3.1)
V or T	82.5	82.2 ± 1.3 (80.3–85.7)	-	-	81.1 ± 0.7 (80.1–81.7)	82.5 ± 1.0 (81.7–83.6)
G1	39.9	46.4 ± 4.8 (39.9–49.5)	-	-	40.7 ± 5.3 (33.6–46.5)	39.5 ± 2.1 (37.8–41.8)
Stylet length	49.5	48.7 ± 2.5 (43.5–51.0)	37.7 ± 1.5 (36.0–39.0)	-	47.1 ± 3.0 (43.5–50.0)	48.0 ± 2.7 (45.0–50.0)
(Stylet length/body length) × 100	11.4	10.9 ± 0.9 (9.1–12.4)	12.2 ± 0.7 (11.6–12.9)	-	11.4 ± 0.7 (10.7–12.4)	11.9 ± 1.4 (11.0–13.4)
Conus length	35.5	34.8 ± 2.3 (28.0–37.0)	27.3 ± 1.2 (26.0–28.0)	-	34.4 ± 2.6 (31.5–37.0)	34.7 ± 2.5 (32.0–37.0)
m	71.7	71.9 ± 0.5 (71.3–72.6)	72.6 ± 1.0 (71.8–73.7)	-	72.9 ± 1.0 (71.7–74.0)	72.2 ± 1.6 (71.1–74.0)
DGO	5.0	6.2 ± 1.2 (5.0–9.0)	5.7 ± 0.6 (5.0–6.0)	-	7.3 ± 0.7 (6.5–8.0)	6.0 ± 1.0 (5.0–7.0)
O	10.1	11.3 ± 2.8 (9.9–19.6)	15.0 ± 1.0 (13.9–15.8)	-	15.5 ± 1.8 (13.0–17.4)	12.5 ± 2.0 (10.2–14.0)
Lip width	7.5	7.6 ± 0.4 (7.0–8.5)	5.3 ± 0.6 (5.0–6.0)	7.0 ± 0.5 (6.5–7.5)	8.1 ± 0.3 (8.0–8.5)	7.2 ± 0.3 (7.0–7.5)
Median bulb length	27.0	25.7 ± 1.8 (23.0–30.0)	-	-	25.0 ± 1.8 (23.0–27.0)	23.3 ± 1.5 (22.0–25.0)
Median bulb width	15.0	12.8 ± 1.5 (11.0–16.0)	-	-	12.0 ± 0.8 (11.0–13.0)	12.0 ± 1.0 (11.0–13.0)
Anterior end to center median bulb	72	71.6 ± 4.1 (62.0–78.0)	-	-	70.5 ± 3.1 (66.0–73.0)	71.7 ± 2.1 (70.0–74.0)
MB	61.5	63.5 ± 3.7 (60.5–74.0)	-	-	68.3 ± 4.7 (62.6–74.0)	62.9 ± 1.0 (61.7–63.8)
Nerve ring to anterior end	91.0	87.2 ± 7.0 (76.0–100.0)	-	-	85.5 ± 4.4 (81.0–91.0)	90.0 ± 1.0 (89.0–91.0)
Excretory pore to anterior end	100.0	103.1 ± 11.7 (80.0–127.0)	82.0 ± 2.0 (80.0–84.0)	90.7 ± 4.7 (87.0–96.0)	97.3 ± 7.5 (90.0–107.0)	106.0 ± 4.4 (103.0–111.0)
Pharynx length	117.0	112.1 ± 10.0 (95.0–122.0)	92.3 ± 2.5 (90.0–95.0)	95.0 ± 7.0 (87.0–100.0)	103.8 ± 9.8 (95.0–115.0)	114.0 ± 2.7 (111.0–116.0)
Maximum body diam.	20.0	21.8 ± 2.9 (18.0–27.0)	20.0 ± 1.0 (19.0–21.0)	21.3 ± 3.3 (14.0–18.0)	20.0 ± 1.6 (18.0–21.5)	22.0 ± 6.2 (17.0–29.0)
Vulva–anus distance	54	55.0 ± 6.1 (41.0–56.0)	-	-	46.3 ± 5.9 (42.0–53.0)	47.7 ± 6.7 (42.0–55.0)
Tail length	34.0	34.1 ± 7.7 (23.0–53.0)	30.0 ± 1.0 (29.0–31.0)	36.0 ± 7.9 (30.0–45.0)	31.1 ± 0.6 (30.5–32.0)	30.0 ± 1.7 (28.0–31.0)
Anal body diam.	13.0	12.6 ± 2.6 (10.0–19.0)	11.7 ± 0.3 (11.5–12.0)	13.7 ± 2.1 (12.0–16.0)	11.5 ± 1.3 (10.0–13.0)	10.7 ± 1.5 (9.0–12.0)

* Abbreviations: a = body length/greatest body diameter; b = body length/distance from anterior end to pharyngo-intestinal junction; DGO = distance between stylet base and orifice of dorsal pharyngeal gland; c = body length/tail length; c’ = tail length/tail diameter at anus or cloaca; G1 = anterior genital branch length expressed as percentage (%) of the body length; L = overall body length; m = length of conus as percentage of total stylet length; MB = distance between anterior end of body and center of median pharyngeal bulb expressed as percentage (%) of the pharynx length; n = number of specimens on which the measurements are based; O = DGO as percentage of stylet length; T = distance from cloacal aperture to anterior end of testis expressed as percentage (%) of the body length; V = distance from body anterior end to vulva expressed as percentage (%) of the body length.

Diagnosis and Relationships

*Paratylenchus plesiostraeleni* sp. nov. is characterized by lateral field with four lines, advulval flap membranes well developed, and a moderately long flexible stylet of 48.7 (43.5–51.0) µm. Lip region continuous with the rest of the body, conoid-rounded, with submedian lobes almost indistinct; and very slight sclerotization; with very slight sclerotization. Spermatheca rounded to spherical. Conoid tail progressively narrowing to form a terminus subacute to finely rounded, corresponding to 0.5–0.8 times as long as the vulva–anus distance. It belongs to group 10 by Ghaderi et al. [2], characterized by a long stylet (>40 µm), lateral field with four lines, and advulval flap membranes.

Morphologically and morphometrically, *P. plesiostraeleni* sp. nov. is almost identical to *P. parastraeleni* and *P. straeleni*, and can be also analogous to *P. goodeyi*. Although general morphology and many matrix codes of *P. plesiostraeleni* sp. nov. by the polytomous key by Palomares-Rius et al. [8] are almost identical to *P. parastraeleni* and *P. straeleni*, both species can be only separated by lip region shape in the lateral view (conoid-rounded, E12 code vs. truncate, anteriorly flattened E4 code, conoid E1 code, respectively) [2,5,8], for all the other characters and matrix codes of all three species are within the same range. Additionally, no considerable differences in morphology and morphometrics can be distinguished among the new species and several *P. straeleni* populations reported from Belgium, Czech Republic, Iran, Italy, Poland, The Netherlands, Turkey, and USA [3,9,19,20,21,22,23]. Consequently, considering the specific molecular markers (D2-D3, ITS and COI) this species could be separated as a new species, this being a valuable illustration of cryptic species within the *P. straeleni*-complex species, and the new species identification can support to delineate the identity of morphometrically related species. *P. goodeyi* can be also separated by lip region shape (conoid-rounded vs. conoid) and c’ ratio (2.8 (2.4–3.3) vs. 1.6–4.9) [2]. According to the polytomous key of Palomares-Rius et al. [8], codes for the new species are (codes in parentheses are exceptions): A3, B3, C3, D1, E12, F2, G3(2), H1, I1(2), J2, K?, L?, M3(2), N3(2), O5(3,4), P?, Q2, R3(2), S2(1), T1, U1(2), V1, W1, X1(2).

Molecular Characterization

Eight D2-D3 of the 28S rRNA (ON873204-ON873211), twelve ITS (ON873174-ON873185), and ten COI gene sequences (ON873954-ON873963) were sequenced for this new species. No intraspecific variation was detected for D2-D3 and ITS, and 98.1% similarity (0–7 nucleotides, 0 indel) was found for COI. Molecularly, the most related species to *P. plesiostraeleni* sp. nov. was *P. nawadus* being 93.0% similar for the D2-D3 region (MN088373) (diverging 52 nucleotides and 1 indel) [16]; *P. nanus* (MH237651) being 92.1% similar (differing 61 nucleotides and 1 indel), and clearly different from the close morphological species *P. parastraeleni* (MZ265064-MZ265067); and *P. straeleni* (MN783711, MW413577-MW413578, MW413685-MW413686) with 90.4–90.7% and 88.7–86.9% similarities (differing 66–67, 73–82 nucleotides and 4 and 5 indels, respectively) [3,5]. ITS region was 81.1–81.3% similar to *P. israelensis* from Spain (MW798343-MW798346), 80.9% similar to *P. neoamblycephalus* from Belgium (MW413606-MW413610), 80.1% similar to *P. sheri* from Spain (MZ265044-MZ265050) (differing in 124 to 125 nucleotides, 130 nucleotides, and 128 to 130 nucleotides, 45 to 47 indels, 59 indels, and 46 indels, respectively) [3,5], and scarce similarity (sequence with a query coverage less than 65%) with the close morphological species *P. straeleni* (MW413625) and *P. parastraeleni* (MZ265005-MZ265007) [3,5]. For COI gene sequences, the similarity values were 90.1–90.6% (differing from 36 to 38 nucleotides and 0 indels) with *P. goodeyi* from Spain (MZ262227-MZ262249), 91.2–90.0% (differing 32 to 38 nucleotides and 0 indel) from *P. veruculatus* from Belgium (MW421720-MW421726) and 90.3% (differing 36 nucleotides and 0 indel) from *P*. *indalus* from Spain (MW797005-MW797008), and clearly different from the close morphological species *P. parastraeleni* (MZ262209-MZ262210) and *P. straeleni* (MN711368, MW421716) 88.0% and 86.7–85.6% (differing 43–46, 50–51 nucleotides and 0 indels, respectively) [3,4,5]. All molecular markers studied clearly separate the new species from other *Paratylenchus* species, including both species being morphologically almost undistinguishable (*P. parastraeleni* and *P. straeleni*).

Type Habitat and Locality

*Paratylenchus plesiostraeleni* sp. nov. was detected in the rhizosphere of maritimus pine (*Pinus pinaster* Ait.), coordinates 36°40′59.2″ N, 4°55′13.3″ W; in the municipal district of Tolox, Malaga province, on the Nieves Mountain, southeastern Spain. Additional specimens of this species were detected in two samples from the same host-plant and two different localities from Bermeja-Crestellina Mountain and Tejeda-Almijara Mountain reported in Table 1.

Etymology

The species epithet, *plesiostraeleni*, is related to a compound name from the Greek word *plesios* = near, and *straeleni*, the morphologically closest species of the genus *Paratylenchus*.

Type Material

Holotype female, 16 paratypes females, 3 third-stage juveniles and 3 fourth-stage juveniles paratypes (slide numbers CMPp4-01, CMPp4-02-CMPp4-10) were maintained in the Nematode Collection of the Institute for Sustainable Agriculture, CSIC, Córdoba, Spain; two females at Istituto per la Protezione delle Piante (IPP) of Consiglio Nazionale delle Ricerche (C.N.R.), Sezione di Bari, Bari, Italy (CMPp4-11); and two females deposited at the USDA Nematode Collection (slide T-7737t).

#### 2.1.3. Remarks of *Paratylenchus canchicus* Mohilal and Dhanachand, 2004, *Paratylenchus caravaquenus* Clavero-Camacho et al., 2021, *Paratylenchus microdorus* Andrássy, 1959, *Paratylenchus nanus* Cobb, 1923, and *Paratylenchus sheri* (Raski, 1973) Siddiqi, 1986

**Figure 7:**
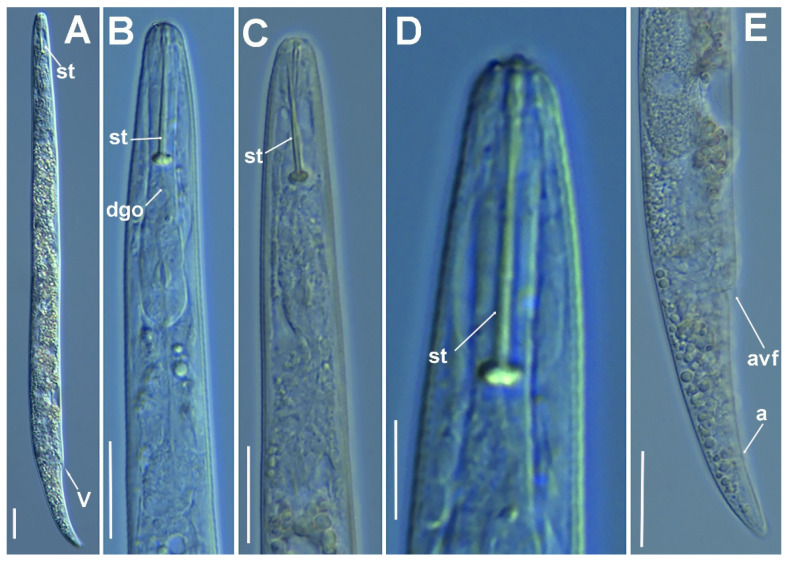
Micro-photomicrographs of *Paratylenchus canchicus* Mohilal and Dhanachand, 2004 female. (**A**) Whole female with vulva arrowed; (**B**,**C**) Pharyngeal region; (**D**) Lip region; (**E**) Female posterior region with vulva and anus (arrowed) and detail of vulva showing advulval flap membrane (arrowed). Scale bars (**A**–**C**,**E** = 20 µm; **D** = 10 µm). (Abbreviations: a = anus; avf = advulval flap; dgo = dorsal gland orifice; st = stylet; V = vulva).

**Figure 8:**
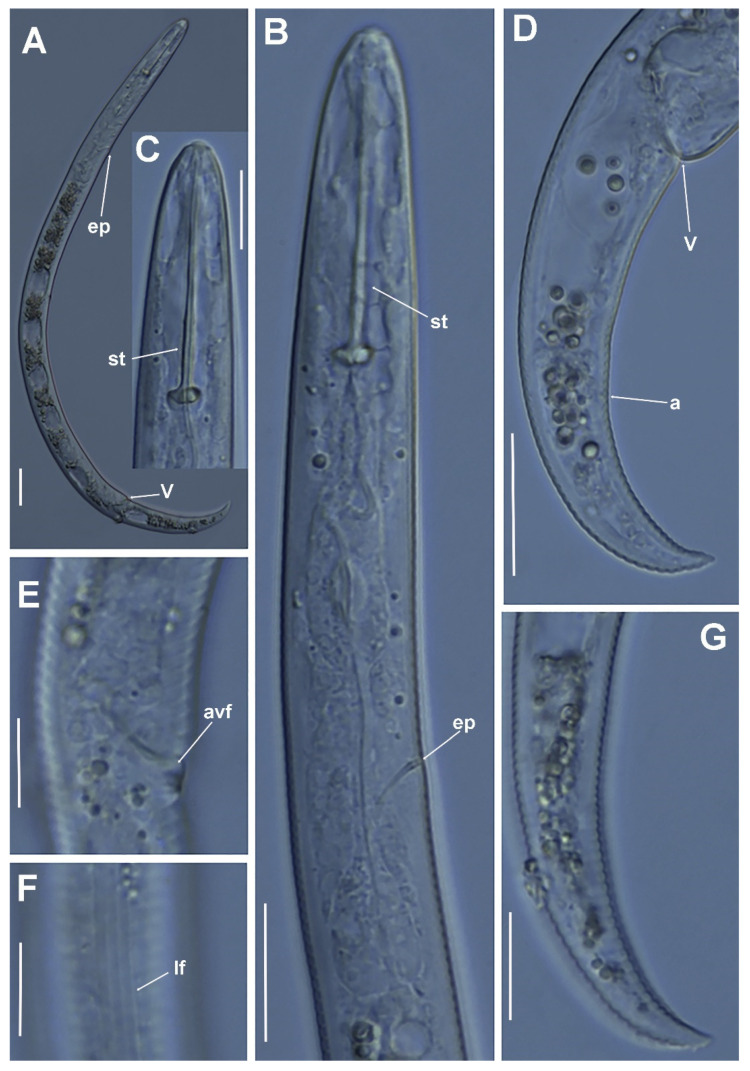
Micro-photomicrographs of *Paratylenchus nanus* Cobb, 1923 female. (**A**) Whole female with excretory pore and vulva arrowed; (**B**) Pharyngeal region with excretory pore arrowed; (**C**) Detail of stylet region; (**D**,**E**) Female posterior region with vulva and anus (arrowed) and detail of vulva showing advulval flap membrane (arrowed); (**F**) detail of lateral fields; (**G**) Detail of female tail. Scale bars (**A**,**B**,**D**,**G** = 20 µm; **C**,**E**,**F** = 10 µm). (Abbreviations: a = anus; avf = advulval flap; ep = excretory pore; lf = lateral field; st = stylet; V = vulva).

(Figure 7 and Figure 8, Table 4).

The Spanish population of *P. canchicus* is characterized by a conoid-rounded lip region, moderate-short stylet, with four lines on the lateral field and advulval flap present, belonging to Group 3 by Ghaderi et al. [2]. Morphology and morphometry of this population is close to original description from Uttar Pradesh, India [24], from which only minor differences were detected in body length (295.8 (281–304) vs. 360–420 µm), which can be associated with the low number of specimens detected and measured in the Spanish population vs. original one (4 vs. 10). This species is morphologically and morphometrically quite close to *P. alleni* [2,25]. However, the available morphological and molecular data on an Iranian population of *P. alleni* (MN168893, annotated in NCBI as Nematoda sp. Dezful, see below) [26] suggest that both species can be a complex of cryptic species; nonetheless, topotype specimens of both species need to be identified by integrative taxonomy to confirm this hypothesis. Thus, these reports are recommended as accepted and referral populations for each species until the topotype material of *P. alleni* and *P. canchicus* becomes available and molecularly characterized. This is the first record for *P. canchicus* in Spain, and represents the second world record after the original description in India [24]. According to the polytomous key of Palomares-Rius et al. [8], codes for the Spanish population of *P. canchicus* are (codes in parentheses are exceptions): A1, B2, C3, D1, E1, F2, G2(1), H2, I1, J1, K?, L?, M2(3), N3, O2(3), P?, Q2, R3, S1(2), T?, U2(1), V1, W1, X1(2), and all of them are identical or within the range for original population [24].

The Spanish population of *P. microdorus* is characterized by a conoid-truncate lip region, moderate-short stylet, with four lines at lateral field and advulval flap present, belonging to Group 3 by Ghaderi et al. [2]. Since this species has been extensively described in our country [4,5], no morphometrical data are provided, but according to the polytomous key of Palomares-Rius et al. [8], codes for the Spanish population of *P. microdorus* are (codes in parentheses are exceptions): A1, B2, C3, D1, E4, F2, G2, H1, I1, J1, K?, L?, M3, N3(4), O4(3), P?, Q2, R3, S1, T3, U1, V1, W1, X2(1), and all of them are identical or within the range for original population [27].

Finally, the Spanish population of *P. nanus* is characterized by a conoid-rounded lip region, moderate stylet, with four lines on the lateral field and advulval flap present, belonging to Group 3 by Ghaderi et al. [2]. The morphology and morphometry of this population are close to original description from North Dakota, USA [28,29], and molecular data confirmed the accurate identification. This species has been already reported in natural mountain grassland at several localities from Granada, southern Spain [30,31], but this is the first molecular identification for Spain. According to the polytomous key of Palomares-Rius et al. [8], codes for the Spanish population of *P. nanus* are (codes in parentheses are exceptions): A2, B2, C3, D1, E1(2), F2, G2, H2, I1, J1, K?, L?, M3, N3(2), O4(5), P?, Q2, R3(2), S1, T?, U2(3), V1, W1, X2, and all of them are identical or within the range for original population [27].

**Table 4:** Morphometrics of *Paratylenchus canchicus* Mohilal and Dhanachand, 2004, *Paratylenchus microdorus* Andrássy, 1959, and *Paratylenchus nanus* Cobb, 1923 females. All measurements are in µm and in the form: mean ± s.d. (range).

	*P. canchicus*	*P. microdorus*	*P. nanus*
	Females	Females	Females
Sample Code	WMPp1	WMPp1	EPPp4
Locality	Casares, Málaga	Casares, Málaga	Carratraca, Málaga
n	4	3	4
L	295.8 ± 10.6 (281–304)	346.0 ± 64.1 (299–419)	422.3 ± 54.1 (388–503)
a*	17.3 ± 1.8 (15.2–19.7)	22.0 ± 2.4 (19.9–24.6)	22.7 ± 0.6 (21.9–23.3)
b	4.0 ± 0.3 (3.7–4.2)	4.1 ± 0.4 (3.7–4.6)	3.7 ± 0.4 (3.5–4.3)
c	13.8 ± 1.8 (11.1–16.0)	13.5 ± 1.3 (12.3–15.0)	16.7 ± 1.9 (15.2–19.3)
c’	2.8 ± 0.4 (2.3–3.1)	3.0 ± 0.2 (2.9–3.2)	3.2 ± 0.3 (2.7–3.4)
V	82.6 ± 0.7 (81.9–83.3)	81.7 ± 0.8 (80.9–82.6)	81.9 ± 0.9 (80.7–82.7)
G1	36.9 ± 5.3 (29.2–40.9)	41.3 ± 5.0 (35.6–44.9)	41.5 ± 4.7 (35.8–47.1)
Stylet length	18.9 ± 0.9 (18.0–20.0)	15.7 ± 0.6 (15.0–16.0)	31.8 ± 1.0 (31.0–33.0)
(Stylet length/body length) × 100	6.4 ± 0.4 (6.1–6.8)	4.6 ± 0.7 (3.8–5.1)	7.6 ± 0.7 (6.6–8.1)
Conus length	11.1 ± 0.6 (10.5–12.0)	11.3 ± 0.6 (11.0–12.0)	24.3 ± 1.0 (23.0–25.0)
m	58.9 ± 2.0 (56.8–61.1)	72.4 ± 3.2 (68.8–75.0)	76.4 ± 1.8 (74.2–78.1)
DGO	4.3 ± 0.5 (4.0–5.0)	2.8 ± 0.6 (2.5–3.5)	6.4 ± 1.1 (5.0–7.5)
O	22.5 ± 1.8 (21.1–25.0)	18.1 ± 3.3 (15.6–21.9)	20.1 ± 3.9 (15.6–24.2)
Lip width	5.3 ± 0.6 (4.5–6.0)	4.3 ± 0.3 (4.0–4.5)	5.6 ± 0.5 (5.0–6.0)
Median bulb length	19.8 ± 1.7 (18.0–22.0)	19.8 ± 0.8 (19.0–20.5)	19.5 ± 2.4 (17.0–22.0)
Median bulb width	7.4 ± 0.5 (7.0–8.0)	7.8 ± 1.4 (7.0–9.5)	9.3 ± 0.5 (9.0–10.0)
Anterior end to center median bulb	38.9 ± 3.3 (36.0–42.0)	44.7 ± 3.8 (42.0–49.0)	65.5 ± 2.1 (63.0–68.0)
MB	52.5 ± 4.2 (48.0–57.6)	52.7 ± 0.5 (52.4–53.3)	58.2 ± 0.9 (57.3–59.5)
Nerve ring to anterior end	51.5 ± 4.7 (47.0–58.0)	59.0 ± 6.2 (54.0–66.0)	80.8 ± 1.0 (80.0–82.0)
Excretory pore to anterior end	63.3 ± 7.0 (53.0–69.0)	77.7 ± 2.1 (76.0–80.0)	87.3 ± 1.7 (85.0–89.0)
Pharynx length	74.3 ± 6.7 (67.0–83.0)	84.7 ± 6.4 (80.0–92.0)	112.5 ± 3.1 (110.0–117.0)
Maximum body diam.	17.3 ± 1.6 (15.0–18.5)	15.7 ± 1.2 (15.0–17.0)	18.6 ± 2.9 (17.0–23.0)
Vulva–anus distance	34.0 ± 2.0 (32.0–36.0)	35.0 ± 2.6 (33.0–38.0)	47.5 ± 8.2 (40.0–58.0)
Tail length	23.8 ± 4.1 (20.0–28.0)	26.0 ± 7.2 (20.0–34.0)	25.3 ± 1.0 (24.0–26.0)
Anal body diam.	8.6 ± 1.5 (6.5–10.0)	8.5 ± 2.2 (7.0–11.0)	8.0 ± 1.1 (7.0–9.5)

* Abbreviations: a = body length/greatest body diameter; b = body length/distance from anterior end to pharyngo-intestinal junction; DGO = distance between stylet base and orifice of dorsal pharyngeal gland; c = body length/tail length; c’ = tail length/tail diameter at anus or cloaca; G1 = anterior genital branch length expressed as percentage (%) of the body length; L = overall body length; m = length of conus as percentage of total stylet length; MB = distance between anterior end of body and center of median pharyngeal bulb expressed as percentage (%) of the pharynx length; n = number of specimens on which measurements are based; O = DGO as percentage of stylet length; V = distance from body anterior end to vulva expressed as percentage (%) of the body length.

Molecular Characterization

Two new populations of *P. caravaquenus* and eight populations of *P. sheri* were also detected in this study. Since both species were recently morphometrical and molecularly characterized [4,5], only sequences from D2-D3 of 28S rRNA were provided, confirming their accurate identification (Table 1), and avoiding data repetition.

Two D2-D3 of 28S rRNA (ON873212-ON873213), and two COI sequences (ON873964-ON873965) were obtained for the first time for *P. canchicus* in this study. In ribosomal genes, no intraspecific variability was detected; however, some molecular variability (0–14 bp, 0 indel) were found between the two COI sequences in this study (ON873964-ON873965). D2-D3 of *P. canchicus* (ON873212-ON873213) showed a low similarity with *P. dianthus* from Taiwan (MN448364), being 90.8% similar (67 bp, 6 indels difference) [32], *P. nanus* from Belgium (MW413575) 89.7% similar (75 bp, 6 indels difference) [3], and *P. tenuicaudatus* from Iran (KU291239) 89.5% similar (76 bp, 4 indels difference) [33], and 86.7% similar (74 bp, 7 indels difference) to *P. alleni* (MN168893) from Iran [26]. Similarly, COI (ON873964-ON873965) showed also a low similarity with *P. straeleni* from Belgium (MW421716) with 88.8% similarity (41 bp, 0 indel difference) [3], *P. veruculatus* from Spain (MW797024-MW797026) with 88.0% similarity (46 bp, 0 indel difference) [5], and *P. goodeyi* from Belgium and Spain (MW421649, MZ262234-MZ262238) with 88.0–88.3% similarity (43 to 46 bp, 0 indel difference) [3,5].

Three D2-D3 of 28S rRNA (ON873230-ON873232), one ITS rRNA (ON873190), and one COI sequences (ON873979) were obtained for *P. microdorus* herein. No intraspecific variability was detected in D2-D3 sequences of *P. microdorus*. D2-D3 of *P. microdorus* (ON873230-ON873232) showed a high similarity with *P. microdorus* from Belgium (MN783712, MW413654-MW413655), being 98.8% similar (8 bp, 3 indels difference) [3], and 96.2% similarity with *P. recisus* from Spain (MZ265119-MZ265120, 26 bp, 1 indels difference) [5]; ITS (ON873190) is also highly similar to *P. microdorus* from Belgium (MN783712, MW413597-MW413600) with a 99.5% similarity (4–5 bp, 1 indel difference) [3], and 92.6% similar to *P. recisus* from Spain (MZ265043) [5]. COI (ON873979) showed interspecific variability with *P. microdorus* from Belgium (MW421666-MW421667) with a 96.5% similarity (13 bp, 0 indel difference) [3], and differing from *P. enigmaticus* from Spain (MZ262222) with a 93.3% similarity (25 bp, 0 indel difference) [5]. Thus, these data confirm the separation of the species complex (*microdorus-recisus-enigmaticus*) on the basis of ribosomal and mitochondrial genes [5].

Finally, only one D2-D3 of 28S rRNA sequence (ON873216) was obtained for *P. nanus* in this study. This sequence showed a high similarity of 99.9% (1 bp, 0 indels difference) with *P. nanus* from Belgium and California, USA (KF242191-KF242195, MW413657-MW413659) [3,9].

#### 2.1.4. Remarks of *Paratylenchus nainianus* Edward & Misra, 1963 and *Paratylenchus neonanus* Mathur, Khan & Prasad, 1967

**Figure 9:**
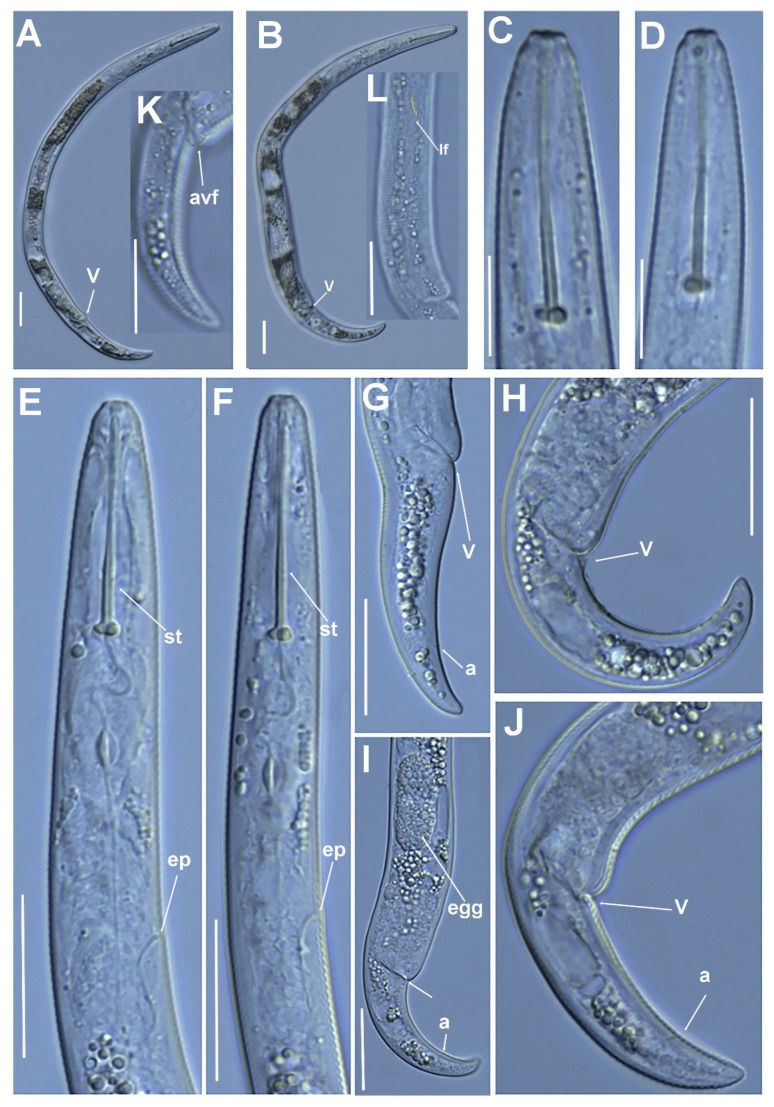
Micro-photomicrographs of *Paratylenchus nainianus* Edward & Misra, 1963 female. (**A**,**B**) Whole female with vulva arrowed; (**C**,**D**) Detail of female stylet region; (**E**,**F**) Pharyngeal region; (**G**–**K**) Female posterior region with vulva and anus (arrowed) and detail of vulva showing advulval flap (arrowed); (**L**) Detail of lateral fields (lines arrowed). Scale bars (**A**,**B**,**E**–**L** = 20 µm; **C**,**D** = 10 µm). (Abbreviations: a = anus; avf = advulval flap; egg = egg, ep = excretory pore; lf = lateral field; st = stylet; V = vulva).

**Figure 10:**
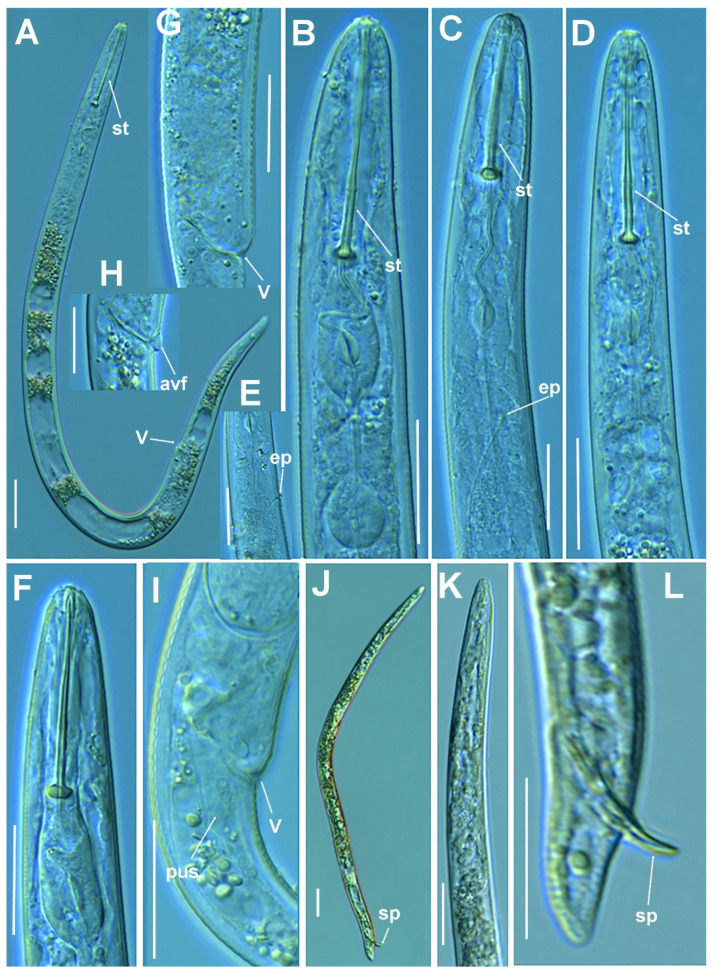
Micro-photomicrographs of *Paratylenchus neonanus* Mathur, Khan & Prasad, 1967 female and male. (**A**) Whole female with stylet and vulva arrowed; (**B–E**) Female pharyngeal region with stylet and excretory pore arrowed; (**F**) Female lip region; (**G–I**) Female posterior region with vulva and Postvulval uterine sac (arrowed) and detail of vulva showing advulval flap (arrowed); (**J**) Whole male; (**K**) Male pharyngeal region showing absence of stylet; (**L**) Male posterior region showing spicules (arrowed). Scale bars (**A–L** = 20 µm). (Abbreviations: avf = advulval flap; ep = excretory pore; lf = lateral field; pus = post-vulval uterine sac; sp = spicules; st = stylet; V = vulva).

(Figure 9 and Figure 10, Table 5).

Two populations of *P. nainianus* were detected in this study, one from maritime pine and another from green heather, both in the same locality (Table 1, Figure 9). The Spanish populations of *P. nainianus* are characterized by a conoid-truncate lip region, moderate-short stylet, distance of the base of median valve to base of stylet knobs 63–72% of the stylet length, female tail terminus rounded, with four lines at the lateral field and advulval flap present, belonging to Group 3 by Ghaderi et al. [2]. This species can be separated from *P. arculatus*, which is already reported in Spain [34] by prominent submedian lobes forming a disc-like structure [2]. Brzeski et al. [34] proposed the synonymization of *P. nainianus* with *P. arculatus*; however, molecular data on the latter species are lacking in order to confirm the synonymization of both species. The morphology and morphometry of these populations are close to the original description from Uttar Pradesh, India [35] and Iran [36]. According to the polytomous key of Palomares-Rius et al. [8], codes for the Spanish populations of *P. nainianus* are (codes in parentheses are exceptions): A2, B3, C3, D1, E4, F2, G1, H1, I1, J1, K?, L?, M1, N2(3), O2(1), P?, Q2, R3(2), S1, T?, U3, V1, W1, X1(2), all of them are identical or within the same range than type population [35].

The Spanish population of *P. neonanus* is characterized by a moderately long stylet (Table 5, Figure 10), lip region conoid-truncate and continuous with body contour, four lateral lines, excretory pore located at isthmus level, advulval flap and post-vulval uterine sac present, spermatheca rounded and filled with sperm, and female tail terminus rounded. A single male was detected for the first time in this species, confirmed by molecular markers (D2-D3 and COI), characterized by a narrower body than a female (Table 5), lessening towards both ends, cuticle finely annulated, with a smooth appearance; lip region analogous to female but slenderer and somewhat truncated, continuous with body, lip region with weak sclerotization, without stylet; pharynx undeveloped and not functional, procorpus, metacorpus, and basal bulb indistinct, outstretched testis, with small sperm, spicule delicate, somewhat bent towards end; gubernaculum slightly curved, without bursa, and short tail, conoid-rounded. According to the polytomous key of Palomares-Rius et al. [8], codes for the Spanish population of *P. neonanus* are (codes in parentheses are exceptions): A2, B2(3), C3, D1, E4, F1, G3, H1, I1, J2, K1, L2, M3(2), N3(2), O4(3), P3, Q2(3), R3(2), S2(1), T?, U2, V1, W1, X1(2), all of them identical or within the same range as type population [37], except for small differences in the c and c’ ratio [37]. The presence of sperm in spermatheca and the first report of the male confirms the amphimictical reproduction of this species.

**Table 5:** Morphometrics of *Paratylenchus nainianus* Edward & Misra, 1963 females and *Paratylenchus neonanus* Mathur, Khan & Prasad, 1967 females and male. All measurements are in µm and in the form: mean ± s.d. (range).

	*P. nainianus*		*P. neonanus*	
	Females	Females	Females	Male
Sample Code	BREE1	WMPp3	WPPp4	WPPp4
Locality	Casares, Málaga		Casares, Málaga	
n	7	10	10	1
L	285.4 ± 24.1 (245–314)	275.7 ± 32.3 (225–308)	365.4 ± 42.6 (314–417)	312
a*	18.6 ± 2.0 (16.5–22.0)	19.2 ± 1.6 (16.9–22.0)	20.9 ± 3.7 (15.7–25.8)	22.3
b	3.8 ± 0.4 (3.4–4.2)	3.5 ± 0.4 (2.9–4.1)	3.7 ± 0.2 (3.4–4.1)	3.7
c	20.0 ± 1.3 (18.8–22.4)	16.7 ± 3.6 (11.2–21.1)	13.8 ± 1.8 (11.1–16.0)	24.0
c’	2.1 ± 0.1 (2.0–2.2)	2.3 ± 0.3 (2.0–3.1)	2.9 ± 0.2 (2.7–3.2)	1.7
V	81.3 ± 0.7 (79.7–82.0)	82.2 ± 1.4 (79.6–84.1)	81.9 ± 1.4 (79.6–84.6)	39.7
G1	39.0 ± 4.5 (33.6–45.2)	39.2 ± 3.0 (35.4–44.9)	38.9 ± 6.5 (29.3–51.4)	-
Stylet length	27.0 ± 1.0 (26.0–29.0)	26.8 ± 1.2 (25.0–28.0)	36.1 ± 1.0 (31.0–37.0)	-
(Stylet length/body length) × 100	9.5 ± 1.1 (8.4–11.8)	9.8 ± 0.9 (8.5–11.1)	10.0 ± 1.0 (8.1–11.5)	-
Conus length	19.2 ± 0.4 (19.0–20.0)	20.5 ± 0.5 (20.0–21.0)	24.7 ± 1.6 (21.0–27.0)	-
m	71.2 ± 1.7 (69.0–73.6)	76.7 ± 3.4 (71.4–84.0)	68.4 ± 4.5 (60.0–75.0)	-
DGO	4.6 ± 0.7 (4.0–6.0)	4.8 ± 0.6 (4.0–6.0)	8.1 ± 0.7 (5.0–9.0)	-
O	17.2 ± 2.8 (14.8–22.6)	17.8 ± 2.6 (14.8–20.4)	11.3 ± 1.9 (7.0–9.0)	-
Lip width	4.4 ± 0.2 (4.0–4.5)	3.7 ± 0.3 (3.5–4.0)	4.8 ± 0.4 (4.0–5.5)	3.5
Median bulb length	19.5 ± 0.5 (19.0–20.0)	18.3 ± 2.1 (14.0–20.0)	20.6 ± 2.5 (18.0–25.0)	-
Median bulb width	8.8 ± 0.4 (8.0–9.0)	7.7 ± 0.5 (7.0–8.5)	9.6 ± 1.3 (7.0–11.0)	-
Anterior end to center median bulb	41.3 ± 3.9 (37.0–48.0)	42.9 ± 2.7 (40.0–46.0)	55.6 ± 5.1 (49.0–64.0)	-
MB	54.3 ± 1.6 (52.1–56.5)	55.0 ± 2.5 (52.3–59.7)	56.9 ± 4.3 (48.6–62.8)	-
Nerve ring to anterior end	52.4 ± 6.7 (46.0–62.0)	53.7 ± 7.4 (43.0–62.0)	72.5 ± 7.4 (65.0–84.0)	-
Excretory pore to anterior end	68.4 ± 4.7 (64.0–78.0)	66.0 ± 7.8 (54.0–74.0)	82.7 ± 10.3 (69.0–96.0)	-
Pharynx length	76.0 ± 5.7 (70.0–85.0)	78.0 ± 4.9 (73.0–87.0)	98.4 ± 12.5 (83.0–116.5)	84
Maximum body diam.	15.5 ± 2.3 (13.0–19.0)	14.4 ± 1.8 (12.0–18.0)	17.8 ± 2.7 (13.0–21.0)	14
Vulva–anus distance	32.0 ± 1.5 (30.0–34.0)	29.8 ± 4.8 (25.0–38.0)	51.7.0 ± 2.5 (49.0–54.0)	-
Tail length	14.3 ± 0.8 (13.0–15.0)	17.1 ± 3.4 (14.0–26.0)	27.0 ± 5.5 (20.0–35.0)	13
Anal body diam.	6.8 ± 0.4 (6.0–7.0)	7.4 ± 0.5 (7.0–8.5)	9.3 ± 1.7 (7.0–11.0)	7.5
	*-*	*-*	-	17.0
	*-*	*-*	-	4.5

* Abbreviations: a = body length/greatest body diameter; b = body length/distance from anterior end to pharyngo-intestinal junction; DGO = distance between stylet base and orifice of dorsal pharyngeal gland; c = body length/tail length; c’ = tail length/tail diameter at anus or cloaca; G1 = anterior genital branch length expressed as percentage (%) of the body length; L = overall body length; m = length of conus as percentage of total stylet length; MB = distance between anterior end of body and center of median pharyngeal bulb expressed as percentage (%) of the pharynx length; n = number of specimens on which measurements are based; O = DGO as percentage of stylet length; T = distance from cloacal aperture to anterior end of testis expressed as percentage (%) of the body length; V = distance from body anterior end to vulva expressed as percentage (%) of the body length.

Molecular Characterization

Six D2-D3 of 28S rRNA (ON873217-ON873222), four ITS rRNA (ON873186-ON873189), and ten COI sequences (ON873966-ON873975) were accomplished for the first time for *P. nainianus* in this study. Low intraspecific variability was detected on D2-D3 sequences (98.3–100.0% similarity, 0–11 bp, 0–2 indels), and no intraspecific variability was detected in ITS; however, some variable positions (from 0 to 15 bp, 0 indel) were found among COI sequences included in this study (ON873966-ON873975). D2-D3 of *P. nainianus* (ON873217-ON873222) showed a low similarity with *P. pedrami* from Spain (MW798283-MW798285) being 83.1% (118–119 bp,16 indels difference) [4], and *P. leptos* from Ethiopia (MW413646-MW413652) with an 83.1% similarity (120 bp, 31 indels difference) [3]. ITS from *P. nainianus* (ON873186-ON873189) showed a scarce similarity (sequence query coverage less than 76%) with *P. minor* from China (MK660189) being 84.6% (79bp, 26 indels) [38] and 84.6% similar to *Paratylenchus* sp. BC (KT258979, 79 bp, 26 indels). Similarly, COI (ON873966-ON873975) showed also a low similarity with *P. baldaccii* from Spain (MZ262220-MZ262221) with an 83.7% similarity (62–64 bp, 0 indel difference) [4], *P. indalus* from Spain (MW797005-MW797008) with an 85.8% similarity (51–53 bp, 1 indel difference) [4], and *P. neonanus* from Spain (ON873975-ON873978) with an 85.6% similarity (55 bp, 0 indel difference).

Four D2-D3 of 28S rRNA (ON873226-ON873229) and three COI sequences (ON873976-ON873978) were obtained for *P. neonanus* for the first time in this study. Very low intraspecific variability was detected in D2-D3 sequences of *P. neonanus* (0–1 bp, 0 indels difference), and no variability was detected in COI sequences. D2-D3 of *P. neonanus* (ON873226-ON873229) showed a low similarity with *P. pedrami* from Spain (MW798283-MW798285) being 84.9% (107 bp, 14 indels difference) [4], and an 84.1–84.6% similarity with *P. baldaccii* from Spain (MW798290-MW798291, MZ265079, 108–111 bp, 14 indels difference) [4,5]. Similarly, COI (ON873976-ON873978) showed a low similarity with *P. caravaquenus* from Spain (MW797003-MW797004) with an 85.0% similarity (54 bp, 0 indel difference) [4], 84.1% (58 bp, 0 indels difference) from *P. aquaticus* B (MW411838) from USA [3], and differing from *P. baldaccii* from Spain (MZ262221) with an 83.8% similarity (61 bp, 2 indels difference) [5].

#### 2.1.5. Remarks of *Paratylenchus salubris* Raski, 1975, *Paratylenchus* sp. 2 SAS and *Paratylenchus wuae* Yu, Ye & Powers, 2016

**Figure 11:**
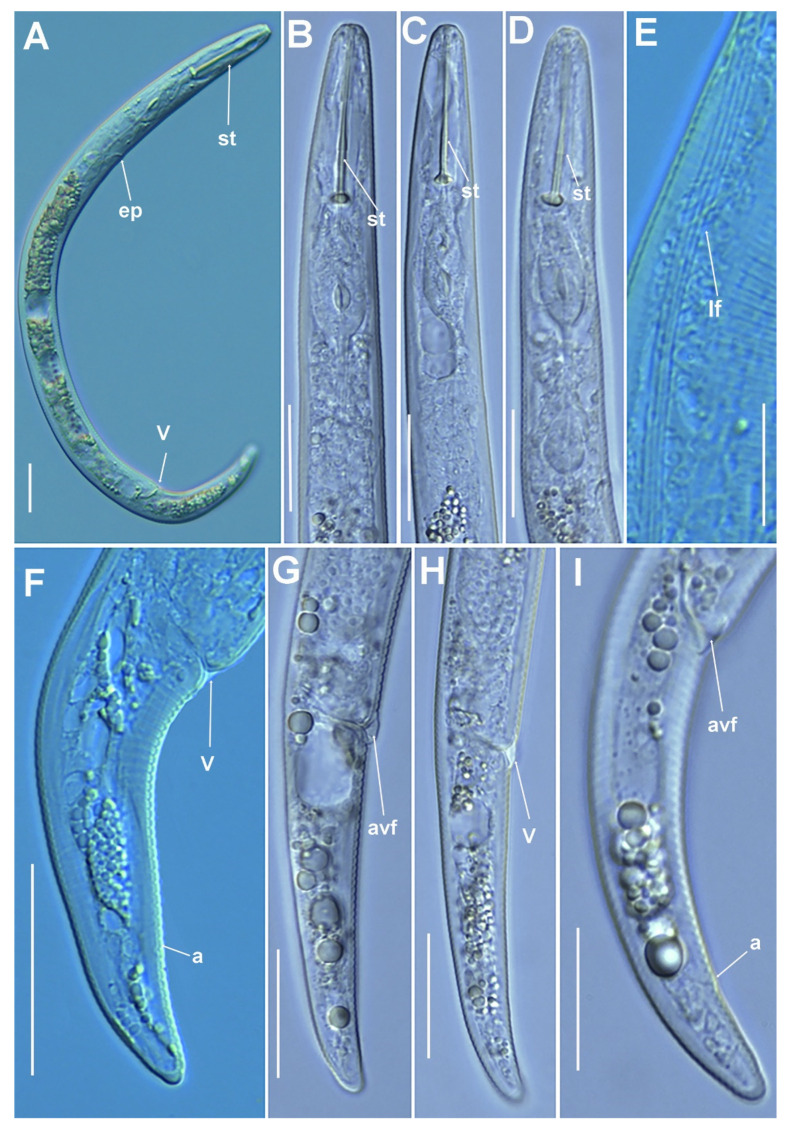
Micro-photomicrographs of *Paratylenchus salubris* Raski, 1975 female. (**A**) Whole female with stylet, excretory pore and vulva arrowed; (**B–D**) Pharyngeal region; (**E**) Detail of lateral fields; (**F**–**I**) Female posterior region with vulva and anus (arrowed) and detail of vulva showing advulval flap (arrowed). Scale bars (**A**–**I** = 20 µm). (Abbreviations: a = anus; avf = advulval flap; ep = excretory pore; lf = lateral field; st = stylet; V = vulva).

**Figure 12:**
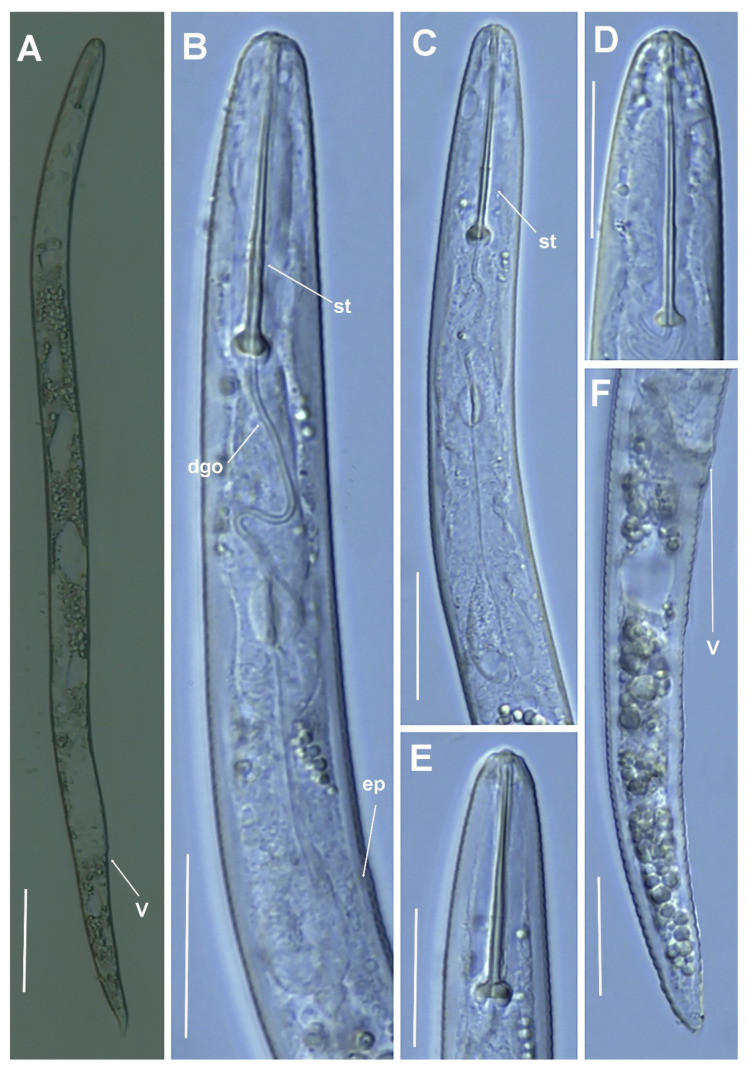
Micro-photomicrographs of *Paratylenchus* sp. 2 SAS female. (**A**) Whole female with vulva arrowed; (**B**,**C**) Pharyngeal region; (**D**,**E**) Detail of female stylet region; (**F**) Female posterior region with vulva (arrowed). Scale bars (**A** = 50 µm; **B–F** = 20 µm). (Abbreviations: dgo = dorsal gland orifice; ep = excretory pore; st = stylet; V = vulva).

**Figure 13:**
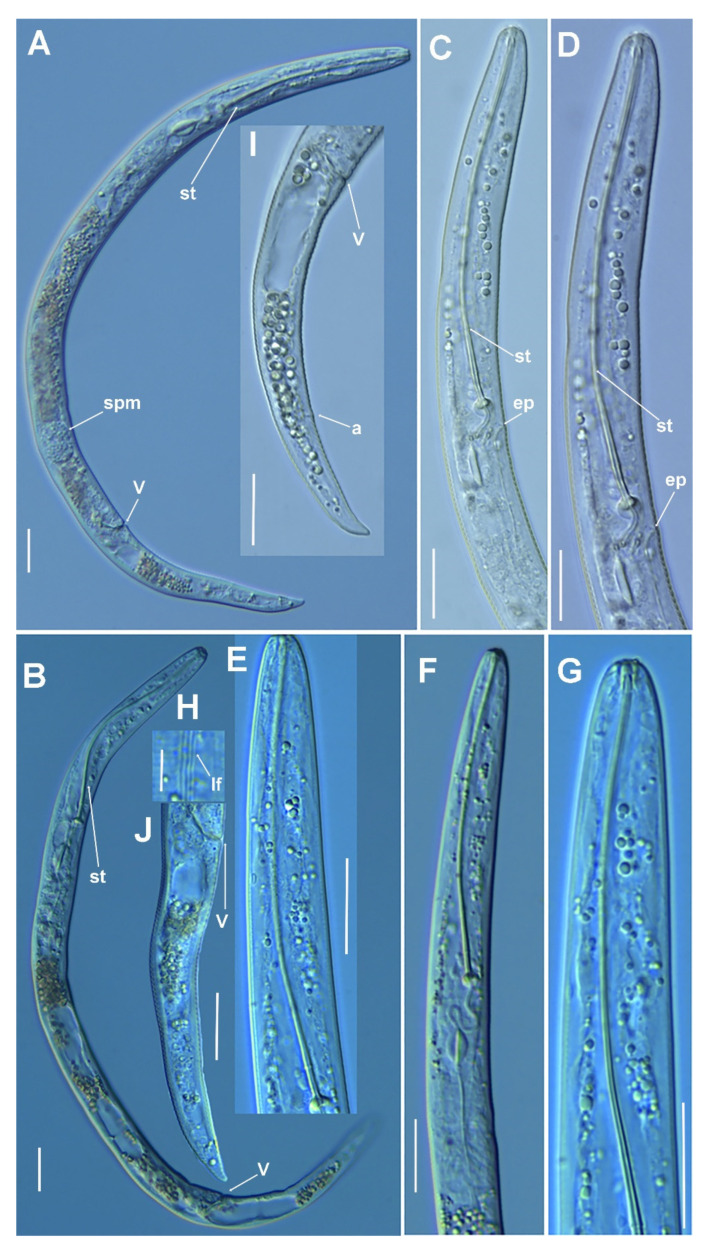
Micro-photomicrographs of *Paratylenchus wuae* Yu, Ye & Powers, 2016 female. (**A**,**B**) Whole female with stylet, vulva and spermatheca arrowed; (**C–G**) Pharyngeal and stylet regions; (**H**) Detail of lateral fields; (**I**–**J**) Female posterior region with vulva and anus (arrowed). Scale bars (**A**–**G**,**I**,**J** = 20 µm; **H** = 10 µm). (Abbreviations: a = anus; ep = excretory pore; lf = lateral field; spm = spermatheca; st = stylet; V = vulva).

(Figure 11, Figure 12 and Figure 13, Table 6).

The Spanish population of *P. salubris* is characterized by a moderately long stylet (Table 6), lip region conoid-rounded, with small submedian lobes and continuous with body contour, four lateral lines, excretory pore located at isthmus level, advulval flap present, spermatheca elongate-oval and filled with sperm, and female tail terminus rounded. Although male specimens were not detected in this study, the presence of sperm in the spermatheca of some females support the amphimictical reproduction of this species. This species has been reported in Brazil and Martinique [25,39], and this is the first report for Spain. According to the polytomous key of Palomares-Rius et al. [8], codes for the Spanish population of *P. salubris* are (codes in parentheses are exceptions): A2, B2, C3, D1, E1(2), F2, G2(1), H2, I1, J2, K?, L?, M1, N2(3), O2(1,3), P?, Q2, R2(3), S1, T?, U3, V1, W1, X1, all of them identical or within the same range than type population [25], except for short differences in body length 244–319 vs. 200–330 µm [2]. Huang and Raski [14] proposed the synonymy of *P. mimulus* with *P. salubris*; however, this action was not accepted by other nematologists [40,41]. Unfortunately, no molecular data of the former species are available to clearly separate both species.

The Spanish population of *Paratylenchus* sp. 2 SAS have a very similar morphology with *P. hamatus* forming a species complex with *Paratylenchus* sp. 1 SAS [3,9]. This population is characterized by a moderate long stylet (Table 6), lip region conoid-rounded, with small submedian lobes and continuous with body contour, four lateral lines, excretory pore located at mid-isthmus and end of basal bulb level, advulval flap present, spermatheca rounded and filled with sperm, and female tail terminus finely rounded. Morphology and morphometrics are coincident with *Paratylenchus* sp. 2 SAS populations from California and Belgium, except for negligible differences in body length (424 (332–486) µm vs. 374 (317–413) µm, 347 (308–389) µm, respectively), stylet length (32.9 (31.5–34.0) µm vs. 29.5 (27.0–33.0) µm, 28.4 (26.5–31.4) µm, respectively), and tail length (31.9 (21.0–41.0) µm vs. 27.5 (22.0–32.0) µm, 26.1 (23.0–28.7) µm, respectively) [3,9]. However, considering the great phenotypic and molecular (ribosomal and mitochondrial) similarity among these three populations from Spain, California and Belgium (see below molecular characterization), all of them need to be considered conspecific. According to the polytomous key of Palomares-Rius et al. [8], codes for the Spanish population of *Paratylenchus* sp. 2 SAS are (codes in parentheses are exceptions): A2, B2, C3, D1, E1(2), F2, G2(3), H2, I1, J2, K?, L?, M3(2), N3(2,4), O4(3,5), P?, Q2, R3(2), S1, T?, U2(1), V1, W1, X2(1), all of them identical or within the same range as Californian and Belgian populations [3,9].

Finally, the two Spanish populations of *P. wuae* belong to Group 11 by Ghaderi et al. [2], and are delineated by a flexible stylet 80.0–94.0 µm long, lip region conoid-rounded, continuous with body contour and prominent submedian lobes, lateral field with four lines, advulval flaps absent, excretory pore located at median bulb level or anterior (just behind stylet knobs), female tail terminus finely rounded, and round to oval spermatheca occupied with sperm, suggesting amphimictic reproduction but males were not detected in this study. Morphometrics of the Spanish populations fit well with type description of *P. wuae* from Ontario, Canada [17] with small differences in the c and c’ ratio (12.6 (11.6–14.1), 3.1 (2.5–3.7) vs. 10.9 (10.5–11.3), 3.2 (3.4–3.8), respectively), and vulva–anus distance (58.7 (54.0–64.0 µm vs. 32 µm), which considering the high molecular (ribosomal and mitochondrial) similarity may be due to geographical intraspecific variability [17]. According to the polytomous key of Palomares-Rius et al. [8], codes for the Spanish populations of *P. wuae* are (codes in parentheses are exceptions): A4, B1, C3, D2, E1(2), F3, G3(2), H1, I3, J2, K?, L?, M4(3), N1, O3(2,4), P?, Q2, R1(2), S2, T?, U1, V1, W1, X1(2), all of them are identical or within the same range than type population [17]. This species was described from Canada and potential undetermined accessions were detected in NCBI (without notifying the country, MW041155, MW041154). Thus, this is the first report for Europe, and an additional example of coincidental pin nematode species between Canada and Spain, where other species such as *P. tateae* or *P. enigmaticus* were also detected in both countries [4,5,42].

**Table 6:** Morphometrics of *Paratylenchus salubris* Raski, 1975, *Paratylenchus* sp. 2 SAS and *Paratylenchus wuae* Yu, Ye & Powers, 2016 females. All measurements are in µm and in the form: mean ± s.d. (range).

	*P. salubris*	*Paratylenchus* sp.2 SAS	*P. wuae*	
	Females	Females	Females	Females
Sample Code	WPPp3	CPPp5	WPPp3	EPPp4
Locality	Casares, Málaga	Tolox, Málaga	Casares, Málaga	Carratraca, Málaga
n	10	11	13	3
L	298.3 ± 21.8 (244–319)	424.0 ± 45.8 (332–486)	350.1 ± 17.0 (326–369)	353.3 ± 14.0 (339–367)
a*	19.5 ± 1.5 (17.4–21.3)	23.1 ± 3.3 (16.4–28.6)	22.7 ± 2.8 (16.4–26.7)	22.1 ± 0.5 (21.6–22.6)
b	3.6 ± 0.3 (3.2–4.0)	4.1 ± 0.6 (3.4–5.5)	2.5 ± 0.2 (2.1–2.8)	2.6 ± 0.1 (2.5–2.7)
c	22.6 ± 2.5 (19.4–27.6)	13.6 ± 2.0 (10.3–17.0)	12.6 ± 0.7 (11.6–14.1)	13.1 ± 0.5 (12.6–13.6)
c’	1.9 ± 0.1 (1.7–2.1)	3.1 ± 0.3 (2.7–3.7)	3.1 ± 0.3 (2.5–3.7)	3.1 ± 0.2 (2.9–3.3)
V	80.6 ± 0.8 (79.3–82.4)	81.2 ± 1.4 (79.7–84.1)	75.8 ± 1.1 (74.7–77.8)	75.7 ± 0.8 (74.9–76.6)
G1	41.1 ± 2.4 (38.6–45.3)	37.3 ± 3.9 (32.4–44.2)	30.4 ± 5.3 (19.9–38.4)	31.2 ± 1.3 (29.7–31.9)
Stylet length	29.3 ± 1.3 (27.0–31.0)	32.9 ± 1.0 (31.5–34.0)	89.4 ± 3.7 (80.0–94.0)	89.5 ± 3.1 (87.0–93.0)
(Stylet length/body length) × 100	9.8 ± 0.6 (9.3–11.1)	7.8 ± 0.9 (6.9–9.8)	25.6 ± 1.9 (21.7–28.5)	25.3 ± 0.3 (25.0–25.7)
Conus length	20.0 ± 1.0 (18.0–21.5)	22.5 ± 1.4 (20.0–24.0)	81.3 ± 3.8 (72.0–85.0)	82.7 ± 2.1 (81.0–85.0)
m	68.2 ± 1.5 (66.7–71.4)	68.3 ± 3.4 (63.5–75.0)	90.9 ± 2.0 (86.7–93.4)	92.4 ± 0.9 (91.4–93.1)
DGO	5.5 ± 0.5 (5.0–6.0)	6.6 ± 1.2 (4.5–9.0)	5.6 ± 1.1 (4.5–8.0)	6.8 ± 1.3 (5.5–8.0)
O	18.6 ± 1.1 (17.2–20.3)	20.1 ± 3.8 (14.1–28.1)	6.2 ± 1.2 (5.1–8.6)	7.6 ± 1.2 (6.3–8.6)
Lip width	4.8 ± 0.3 (4.5–5.0)	4.9 ± 0.5 (4.0–5.5)	5.0 ± 0.4 (4.5–6.0)	4.7 ± 0.3 (4.5–5.0)
Median bulb length	18.0 ± 2.8 (12.0–22.0)	23.1 ± 2.0 (20.0–25.0)	21.7 ± 1.2 (20.0–24.0)	21.0 ± 1.0 (20.0–22.0)
Median bulb width	8.6 ± 0.8 (7.5–10.0)	10.1 ± 0.7 (9.0–11.0)	9.6 ± 0.6 (9.0–11.0)	9.5 ± 0.5 (9.0–10.0)
Anterior end to center median bulb	46.7 ± 2.9 (43.0–52.0)	60.6 ± 7.8 (47.0–67.0)	104.1 ± 4.4 (95.0–111.0)	102.0 ± 3.5 (98.0–104.0)
MB	56.0 ± 3.9 (45.8–59.9)	57.4 ± 2.2 (53.4–60.6)	74.6 ± 5.6 (65.1–86.1)	77.3 ± 1.2 (76.5–78.2)
Nerve ring to anterior end	59.8 ± 3.7 (54.0–66.0)	77.5 ± 11.9 (51.0–91.0)	119.4 ± 5.4 (110.0–130.0)	116.3 ± 3.8 (112.0–119.0)
Excretory pore to anterior end	72.1 ± 4.2 (53.0–69.0)	94.5 ± 10.1 (79.0–106.0)	99.5 ± 6.2 (91.0–109.0)	94.7 ± 4.0 (91.0–99.0)
Pharynx length	74.3 ± 6.7 (63.0–77.0)	105.4 ± 10.8 (85.0–115.0)	140.2 ± 9.7 (122.0–154.0)	133.7 ± 2.1 (132.0–136.0)
Maximum body diam.	15.4 ± 1.1 (14.0–17.5)	18.8 ± 4.1 (14.0–29.0)	15.7 ± 2.0 (13.5–21.0)	16.0 ± 1.0 (15.0–17.0)
Vulva–anus distance	39.8 ± 6.4 (32.0–47.0)	53.1 ± 9.4 (43.0–66.0)	58.7 ± 5.0 (54.0–64.0)	62.0 ± 2.0 (60.0–64.0)
Tail length	13.3 ± 1.3 (10.5–13.0)	31.9 ± 6.0 (21.0–41.0)	27.8 ± 1.7 (26.0–31.5)	27.0 ± 1.0 (26.0–28.0)
Anal body diam.	7.0 ± 0.5 (6.0–7.5)	10.2 ± 1.8 (7.0–13.0)	8.9 ± 0.7 (8.0–11.0)	8.8 ± 0.3 (8.5–9.0)

* Abbreviations: a = body length/highest body diameter; b = body length/distance from anterior end to pharyngo-intestinal junction; DGO = distance between stylet base and orifice of dorsal pharyngeal gland; c = body length/tail length; c’ = tail length/tail diameter at anus or cloaca; G1 = anterior genital branch length expressed as percentage (%) of the body length; L = overall body length; m = length of conus as percentage of total stylet length; MB = distance between anterior end of body and center of median pharyngeal bulb expressed as percentage (%) of the pharynx length; n = number of specimens on which measurements are based; O = DGO as percentage of stylet length; V = distance from body anterior end to vulva expressed as percentage (%) of the body length.

Molecular Characterization

Three D2-D3 of 28S rRNA (ON873233-ON873235) and four COI sequences (ON873980-ON873983) were accomplished for the first time for *P. salubris*. No intraspecific variability was detected on D2-D3 and COI sequences included in this study. D2-D3 of *P. salubris* (ON873233-ON873235) showed a limited similarity with *P. pedrami* from Spain (MW798283-MW798285), being 96.1% (27–28 bp, 0 indels difference) [4], 94.7–94.8% (36–37 bp, 0 indels difference) from *P. baldacci* from Spain [4], and 85.3–85.9% (100–104 bp, 16–18 indels difference) from *P. leptos* from Ethiopia (MW413646-MW413652) [3]. Similarly, COI (ON873980-ON873983) showed also a low similarity with *P. baldaccii* from Spain (MW797012, MZ262220-MZ262221) [4,5], and *P. pedrami* from Spain (MW797009) with 89.0–89.2% similarities (41–42 bp, 4 indels difference) and 86.1% similarity (51 bp, 2 indels difference) [4], respectively.

Four D2-D3 of 28S rRNA (ON873242-ON873245), three ITS rRNA (ON873191-ON876193), and four COI sequences (ON873985-ON873987) were accomplished for *Paratylenchus* sp. 2 in this study. No intraspecific variability was detected in ribosomal and mitochondrial sequences of *Paratylenchus* sp. 2. D2-D3 of *Paratylenchus* sp. 2 (ON873242-ON873245) from Spain were almost identical (99.0%, 7 bp, 0 indels difference) with *Paratylenchus* sp. 2 (KF242221, MW413670-MW413671) from USA and Belgium [3,9], and also with a high similarity with *P. hamatus* from Spain (OL884394-OL884395), being 98.0% (14 bp, 1 indel difference) [7], and 97.1% similarity with *P. tenuicaudatus* from Iran and Spain (KU291239, OL884408, 20–21 bp, 0 indels difference) [7,33]; ITS (ON873191-ON876193) was also similar to *Paratylenchus* sp. 2 from Belgium (MW413616) with a 96.5% similarity (28 bp, 2 indels difference) [3], and being 97.0% (23–26 bp, 1 indel difference) similar to *P. hamatus* from USA and Spain (KF242247-KF242258, MW798340-MW798341) [5,9]. Similarly, COI (ON873985-ON873987) was also similar to *Paratylenchus* sp. 2 from Belgium (MW413683-MW421685) 96.8–97.0% (11–13 bp, 0 indels difference) [3], and 95.7% similar to *P. hamatus* from Spain (MW797017, 16 bp, 0 indels difference) [4]. Thus, COI sequences confirm the species separation between *Paratylenchus* sp. 2 and *P. hamatus* [3], and supported the hypothesis that the latter species is associated with fruit trees [3,7], whereas the former was only detected on several grasses (including *Salix* sp. And other grasses), and never detected on fruit-trees [7,9]. In any case, additional studies with a wide set of populations from several geographical areas are needed to clarify this species complex.

Five D2-D3 of 28S rRNA (ON873247-ON873251), two ITS rRNA (ON873194-ON876195), and seven COI sequences (ON873988-ON873994) were accomplished for *P. wuae* in this study. Low intraspecific variability in D2-D3 and COI (0–2 bp, 1 bp, 0 indels difference, respectively) and no intraspecific variability on ITS sequences of *P. wuae* was detected in this study. D2-D3 of *P. wuae* (ON873247-ON873251) were almost identical (99.6% similarity, 3 bp, 0 indels difference) with type material of *P. wuae* (KM061782) from Canada [17], and a high similarity with *P. macrodorus* from Spain (MZ265109-MZ265111), being 99.3–99.4% (4–5 bp, 0 indel difference) [5], 99.0% (7–8 bp. 0 indels difference) similar to *P. pandatus* from Spain (MZ265116-MZ265117) [5], but highly different with 92.0–92.2% similarity with *P. vitecus* from Spain (MZ265137-MZ265140, 56–57 bp, 0 indels difference) [5]. ITS (ON873194-ON876195) was also highly similar to the type material of *P. wuae* from Canada (KM061783) with a 99.5% similarity (4 bp, 2 indels difference) [17], 96.5–96.7% (15–28 bp, 7–9 indel difference) similar to *P. macrodoratus* from Spain (MZ265034-MZ265038) [5], also similar, but with low sequence coverage (64–80%), with *P. pandatus* (MZ265041-MZ265042, 8–15 bp, 2–5 indels difference), and *P. peraticus* (MK506792, 71 bp, 5 gaps) from Spain and Iran, respectively [5,43]. Similarly, COI (ON873988-ON873994) was also highly similar to *P. wuae* from China and Canada (MF770965-MF770966, MN710985) 98.9.0% (4 bp, 0 indels difference) [18,44], but different to other species, such as, 94.1–94.4% (21–22 bp, 0 indels difference) similar to *P. macrodorus* from Spain [5], 92.6–93.2% (28–29 bp, 0 indels difference) similar to *P. pandatus* from Spain [5], and 91.6% similar to *P. vitecus* from Spain (MZ262272, 31 bp, 0 indels difference) [5]. These data support that *P. wuae*, *P. macrodoratus* and *P. pandatus* comprise a species complex that can be separated by some morphological characters (*viz.* advulval flap, lip region shape, submedian lobes, shape of spermatheca, vulva-anus distance) and by COI sequences [5,8].

### 2.2. Phylogenetic Analyses of Paratylenchus Species

**Figure 14:**
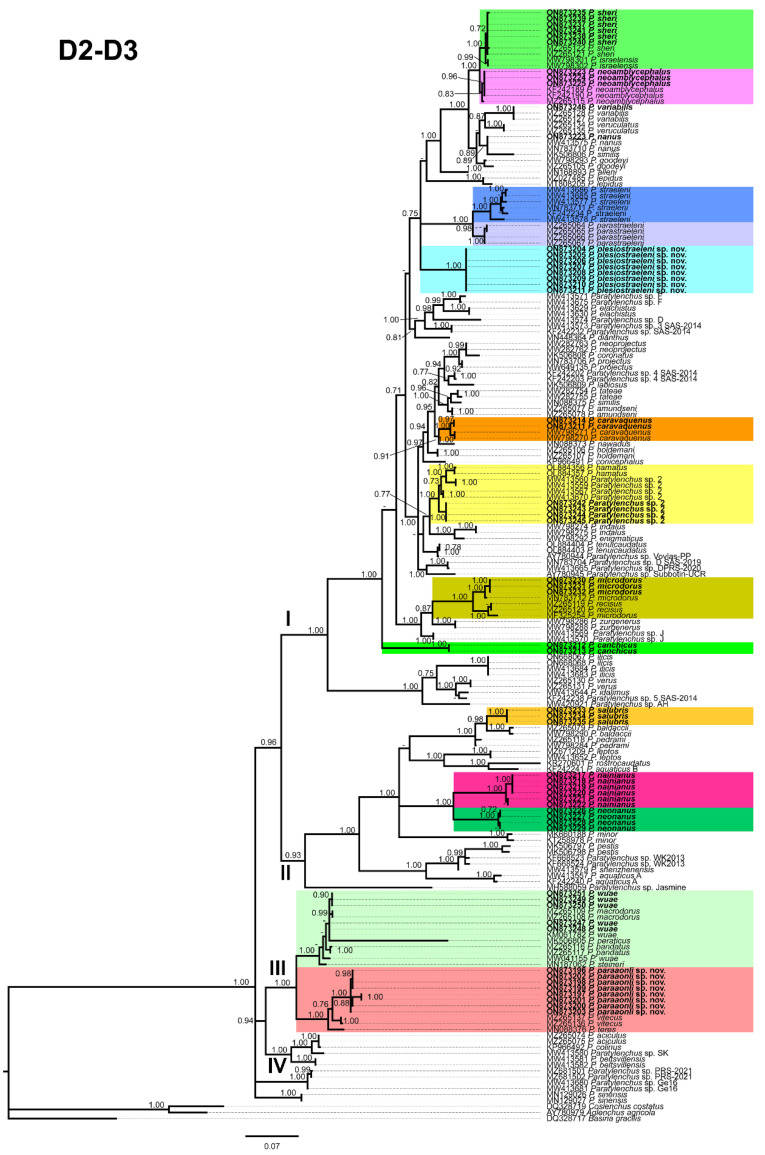
Phylogenetic relationships within the genus *Paratylenchus*. Bayesian 50% majority rule consensus tree as completed from D2-D3 expansion domains of the 28S rRNA sequence alignment under the general time-reversible model with invariable sites and gamma distribution model (GTR + I + G). Posterior probabilities of more than 0.70 are given for appropriate clades. Newly obtained sequences in this study are shown in bold. The scale bar indicates expected changes per site, and the colored boxes indicate the clade association of new *Paratylenchus* species sequenced in this study.

**Figure 15:**
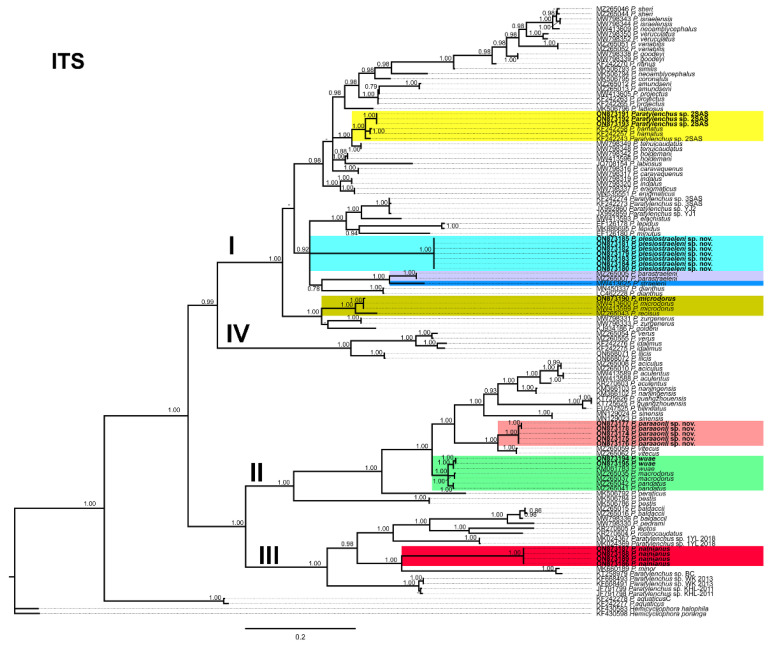
Phylogenetic relationships within the genus *Paratylenchus*. Bayesian 50% majority rule consensus tree as completed from ITS rRNA sequence alignment under the general time-reversible model with invariable sites and gamma distribution model (GTR + I + G). Posterior probabilities of more than 0.70 are given for appropriate clades. Newly obtained sequences in this study are shown in bold. The scale bar indicates expected changes per site, and the colored boxes indicate the clade association of new *Paratylenchus* species sequenced in this study.

**Figure 16:**
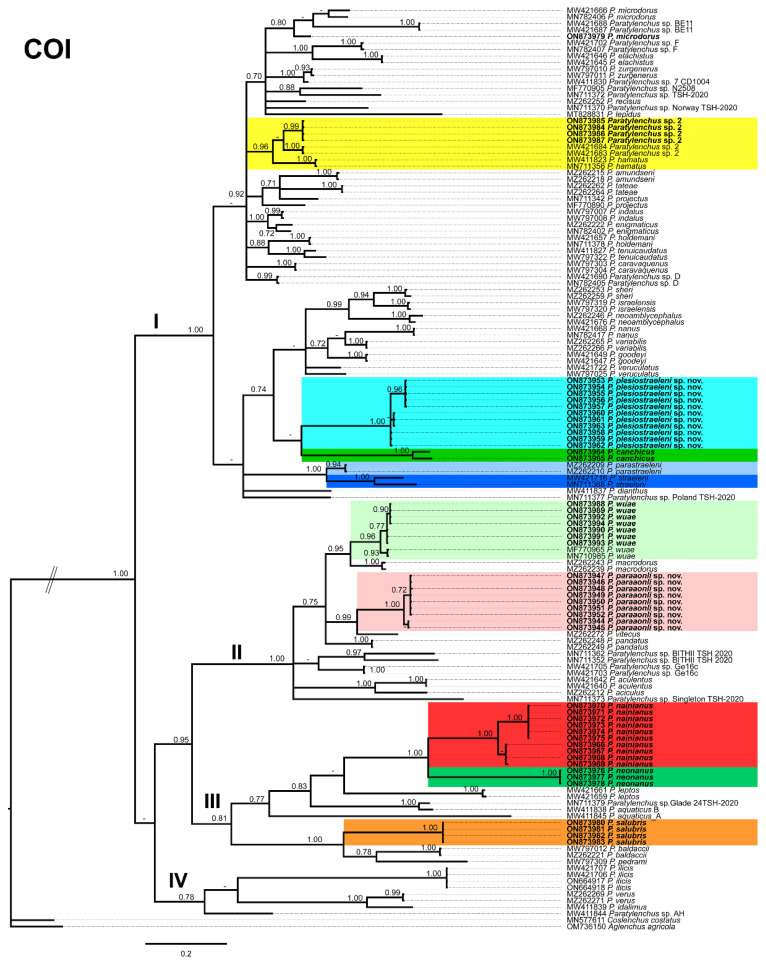
Phylogenetic relationships within the genus *Paratylenchus*. Bayesian 50% majority rule consensus tree as completed from cytochrome c oxidase subunit 1 (COI) sequence alignment under the general time-reversible model with invariable sites and gamma distribution model (GTR + I + G). Posterior probabilities of more than 0.70 are given for appropriate clades. Newly obtained sequences in this study are shown in bold. The scale bar indicates expected changes per site, and the colored boxes indicate the clade association of new *Paratylenchus* species sequenced in this study.

Phylogenetic relationships among *Paratylenchus* species completed from analyses of D2-D3 domains of the 28S rRNA, ITS rRNA, and COI gene sequences using Bayesian inference (BI) are given in Figure 14, Figure 15 and Figure 16, respectively. The D2-D3 of the 28S rRNA gene alignment (705 bp long) comprised 185 sequences with 77 *Paratylenchus* species and three outgroup species (*Basiria gracillis* (DQ328717), *Aglenchus agricola* (AY780979), and *Coslenchus costatus* (DQ328719). Fifty-six new sequences were contained in this analysis. The Bayesian 50% majority rule consensus tree completed from the D2-D3 alignment is given in Figure 14, and contained three well- (I, III, IV) and one (II) moderately-supported clade (PP = 1.00, PP = 0.93, respectively, Figure 14). Clade I grouped 49 species mostly with short and unbending stylet < 40 µm and conus about 50% of the total stylet, but also some longer stylet species (i.e., *P. straeleni*-species complex, *P. goodeyi*), including 43 species of the morphospecies Group 3, 1 species from Group 8, 4 species from Group 10, and 1 species from Group 11 [2]. *Paratylenchus plesiostraeleni* sp. nov. grouped in a separate subclade from *P. straeleni* and *P. parastraeleni*, but all of them within a low supported clade (PP = 0.75) with several new sequenced species with shorter stylet *viz*. *P. sheri*, *P. neoamblycephalus*, *P. variabilis*, *P. nanus*, *P. caravaquenus*, *Paratylenchus* sp. 2, *P. microdorus*, and *P. canchicus* (Figure 14). Clade II grouped 14 species belonging also to several morphospecies groups, including 8 species from Group 3, 3 species from Group 2, 2 species from Group 4, and 1 species from Group 11 [2]. Clade III grouped 8 species with a long and flexible stylet > 40 µm with conus corresponding to about more than 70% of the total stylet, belonging to Group 10 (3 species, including *P. paraaonli* sp. nov.), and Group 11 (5 species, including *P. wuae*) (Figure 14) [2]. Additionally, clade IV grouped 4 species belonging to Group 8 (2 species), Group 9 (1 species), and 1 undetermined species (Figure 14) [2]. These clades are primarily equivalent with previous studies on *Paratylenchus* spp. phylogeny [3,4,5,6,9,45].

The ITS rRNA gene alignment (777 bp long) comprised 120 sequences with 59 *Paratylenchus* species and two outgroup species (*Hemicycliophora halophila* (KF430583), and *H. poranga* (KF430598)). Twenty-two new sequences were analyzed in this phylogeny. The Bayesian 50% majority rule consensus tree completed from the ITS alignment is given in Figure 15 and comprised four (I-IV) well-supported clades (PP = 1.00, Figure 15). Clade I grouped 32 species mostly with short and rigid stylet < 40 µm belonging to morphospecies Group 3 [2], but also 4 species with longer stylet (*viz*. *P. plesiostraeleni* sp. nov., *P. parastraeleni*, *P. straeleni*, and *P. goodeyi*, from Group 10, Figure 15) [2]. *Paratylenchus plesiostraeleni* sp. nov. also grouped in a separate subclade from *P. straeleni* and *P. parastraeleni*, but all of them within a moderately supported clade (PP = 0.92) together with several species with shorter stylet *viz*. *P. dianthus*, *P. elachistus*, *P. lepidus*, *P. minutus*, and *Paratylenchus* sp. 3 (Figure 15). Clade II grouped 14 species, all of them with long and flexible stylet > 40 µm, belonging to Group 7 (1 species), Group 8 (1 species), Group 9 (2 species), Group 10 (4 species, including *P. paraaonli* sp. nov.), and Group 11 (6 species). Similar to D2-D3 tree, *P. paraaonli* sp. nov. clustered with *P. vitecus* in a well-supported subclade (PP = 1.00), and *P. wuae* from Spain (ON873194-ON873195) clustered with *P. wuae* type population from Canada [17], and *P. macrodorus* and *P. pandatus* from Spain [5], in a well-supported subclade (PP = 1.00), but well separated from *P. peraticus* from Iran [43] (Figure 15). Clade III grouped 9 species with short stylet (< 40 µm), but belonging to several morphospecies groups, Group 3 (6 species), Group 2 (2 species) and Group 4 (1 species). The newly sequenced *P. nainianus* clustered with *P. minor* and *Paratylenchus* sp. BC in a well-supported subclade (PP = 1.00). Finally, clade IV grouped three species with long and flexible stylet > 40 µm, including *P. verus*, *P. idalimus*, and *P. ilicis*, in a well-supported clade (Figure 15). These clades are primarily equivalent with other latter studies on *Paratylenchus* spp. phylogeny [3,4,5,6,9].

COI gene alignment (421 bp long) comprised 140 sequences with 60 *Paratylenchus* species. In previous studies, three *Hemicycliophora* species were selected as outgroups [4,5]; however, in order to cover the great variability of the present dataset, *Aglenchus agricola* (OM736150) and *Coslenchus costatus* (MN577611) were selected as outgroups in this research. Fifty-one new sequences were in this phylogeny. The Bayesian 50% majority rule consensus tree completed from the COI sequence alignment is given in Figure 16 and is composed of two well-supported clades (I, II) (PP = 1.00, PP = 1.00), and two (III, IV) low-supported clades (PP = 0.81, PP = 0.78, respectively) (Figure 16). Clade I grouped 37 species mostly with short and rigid stylet < 40 µm belonging to morphospecies Group 3 [2], but also 4 species with longer stylet (*viz*. *P. plesiostraeleni* sp. nov., *P. parastraeleni*, *P. straeleni*, and *P. goodeyi*, from Group 10, Figure 16) [2]. *Paratylenchus plesiostraeleni* sp. nov. grouped with eight species of short stylet (including *P. canchicus*) in a low supported subclade (PP = 0.74), but clearly separated from the subclade of *P. straeleni* and *P. parastraeleni* (Figure 16). Additionally, *P. hamatus* and *Paratylenchus* sp. 2 clustered with previous sequences of these species (Figure 16). Clade II grouped 11 species, most of them with long and flexible stylet > 40 µm, belonging to Group 9 (2 species), Group 10 (1 species, including *P. paraaonli* sp. nov.), and Group 11 (8 species, including *P. wuae*). As shown in ribosomal trees (Figure 14 and Figure 15), *P. paraaonli* sp. nov. also clustered with *P. vitecus* in a well-supported subclade (PP = 0.99); and *P. wuae* from Spain (ON873988-ON873994) clustered together with *P. wuae* from a type of population from Canada [17] and *P. macrodorus* from Spain [5] in a well-supported subclade (PP = 0.95) (Figure 16). Clade III grouped 8 species with short stylet (< 40 µm) and belonging to morphospecies groups Group 3 (5 species including *P. nainianus* and *P. neonanus* in a well-supported subclade, PP = 1.00), and Group 2 (3 species) [2]. Finally, clade IV grouped four species with long and flexible stylet > 40 µm, including *P. ilicis*, *P. verus*, and *P. idalimus*, in a low-supported clade (PP = 0.78, Figure 16). These clades are primarily equivalent with other latter studies on *Paratylenchus* spp. phylogeny [3,4,5,6,9].

## 3. Discussion

This study intends to decipher the biodiversity of pin nematodes in mountainous natural environments in Southern Spain and complements other studies mainly related to cultivated and wild areas, demonstrating the existence of the cryptic diversity of this group of nematodes [4,5]. We found 27 Spanish populations of *Paratylenchus* spp. in the rhizosphere of maritimus pine and green heather, from which we identified fourteen species, two of them are described herein as new species (*P. paraaonli* sp. nov., *P. plesiostraeleni* sp. nov.), six of them were first reports for Spain (*P. canchicus*, *P. nainianus*, *P. neonanus*, *P. salubris*, *Paratylenchus* sp. 2 SAS, and *P. wuae*), and six species (*P. caravaquenus*, *P. microdorus*, *P. nanus*, *P. neoamblycephalus*, *P. sheri*, and *P. variabilis*) have been already reported in our country and characterized under integrative taxonomical approaches [4,5]. Consequently, these data increase the biodiversity of pin nematodes in Spain comprising a total of 47 species (33.1% out of 142 total species), from which only 8 species have not been molecularly characterized in Spain (*viz*. *P. aonli*, *P. arculatus*, *P. ciccaronei*, *P. mirus*, *P. projectus*, *P. steineri*, *P. straeleni*, and *P. vandenbrandei*), and need to be completed in order to clarify if these morphological identifications harbor new cryptic diversity. Interestingly, some species expand their distribution geographically (i.e., *P. caravaquenus* and *P. sheri*), considering only species with available molecular data and identified using an integrative approach in Spain [4,5,7]. Some of these species shared cultivated and wild habitats, indicating that the ecological requirements are different, and can be due to the importation of nematode individuals with soil movement between regions/countries or by other means to the cultivated areas. Surprisingly, some species with molecular data available and identified using an integrative taxonomy are detected in different continents under wild habitats (USA-Spain, *Paratylenchus* 2 SAS) [9] or under cultivated (Canada) [17] vs. wild environments in two locations in Spanish forests for *P. wuae*. This could raise the point of a possible introduction of *P. wuae* from wild environments to cultivated environments in other countries and their adaptation or this species occupied a former wider distribution in the planet. In any case, upon the present results, new studies on wild environments in Spain are needed to unravel the actual biodiversity of these nematodes and corroborate if this area is a hotspot of biodiversity as previously suggested [4,5]. Cryptic speciation has frequently been described within pin nematodes, subsequently these data enhanced the hypothesis that the genus *Paratylenchus* may be a hyperdiverse group of nematodes [3,4,5,6,7,9], although further surveys are needed to validate this point.

The specific identification of *Paratylenchus* spp. is also problematic by the presence of several *Paratylenchus* species within the same soil sample, particularly in wild and cultivated environments [4,5]. In this research we recognized the presence of up to three *Paratylenchus* species (*viz*. *P. paraaonli* sp. nov., *P. plesiostraeleni* sp. nov., *P. neonanus*) within the same sample in several cases (Table 1), corroborating the need for developing molecular markers to support this laborious task. Additionally, these nematodes showed a great phenotypic plasticity with limited species-specific diagnostic characters. Recent studies confirmed the prerequisite of using ribosomal and mitochondrial markers for an accurate identification under integrative taxonomical approaches [3,4,5,6,7,8,9,42]. Morphological studies integrated with ribosomal and mitochondrial markers (D2-D3 expansion domains of the 28S rRNA gene, ITS rRNA gen, and mtDNA gene COI) are imperative tools for precise identification of *Paratylenchus* spp. and deciphering the cryptic diversity of pin nematodes in a complex scenario such as natural environments and give unequivocal molecular markers associated with a specific morphology–morphometry for species identification. Our data support also that *P. straeleni*-complex species with three recognized species (*P. straeleni*, *P. parastraeleni* and *P. plesiostraeleni* sp. nov.) and several putative undescribed species comprise a prototypical case of morphostatic speciation (that is, genetic modifications not reproduced in morpho-anatomy) [3,4,5,6,9], since independent methods based on molecular analyses by means of ribosomal and mitochondrial sequence data clearly separate the *P. straeleni*-complex species.

The intraspecific ribosomal sequence variability (D2-D3 and ITS rRNA gene) of *Paratylenchus* species identified in this study was low (ranging from 0 to 11 bp and 0 indels, 98.3–100% similarity, 0–7 bp and 0 indels, 98.1–100% similarity, respectively); although intraspecific mitochondrial variability (COI) was moderate, ranging from 0 to 15 bp and 0 indels, 96.1–100%. These results are within the variability range of *Paratylenchus* species established in a recent study by Palomares-Rius et al. [8] and agree with the hypothesis of faster coalescence within species linages in mitochondrial than nuclear markers [3,46,47]. Furthermore, the absence of intraspecific variability in ribosomal and mitochondrial markers in several *Paratylenchus* species (*viz. P. plesiostraeleni* sp. nov., *P. canchicus*, *P. microdorus*, *P. neoamblycephalus*, *P. neonanus*, *P. salubris*, *Paratylenchu*s sp. 2), may suggest a continuous isolation of these populations under the same natural environmental conditions maintaining biological (host-plants) and ecological traits (soil, temperature, etc.), similar to other occurring situations in criconematids [48].

Phylogenetic analyses constructed on D2-D3, ITS, and partial COI using BI give rise to a consistent position for the new *Paratylenchus* species from Spain described herein (*P. plesiostraeleni* sp. nov., *P. paraaonli* sp. nov.), which were grouped in a separated subclade as a valid species from the *P. straeleni*-complex species (including *P. straeleni* and *P. parastraeleni*), and *P. paraaonli* sp. nov. clustered with *P. vitecus*, but clearly separate from this species. Ribosomal and mitochondrial phylogenies essentially agree with the clustering achieved by other nematologists [3,4,5,9]. As indicated in phylogenetic results and in previous reports by several authors, ribosomal and mitochondrial phylogenies confirm that flexible and long stylet length species (> 40 µm, initially belonging to ‘*Gracilacus*’ or ‘*Cacopaurus*’) and rigid short stylet length species (< 40 µm, initially belonging to ‘*Paratylenchus s.s.*’) cannot be separated in consistent clades, suggesting several convergent evolution events for this trait [3,4,5,6,9].

Finally, the present results emphasized former results on the noteworthy biodiversity of several genera of plant-parasitic nematodes in southern Spain, such as species within the family Longidoridae (including virus vector nematodes of the genera *Xiphinema* and *Longidorus*) or pin nematodes of the genus *Paratylenchus* [4,5,49], and warranty supplementary sampling efforts to elucidate the actual biodiversity in Spain.

## 4. Materials and Methods

### 4.1. Sampling Sites and Nematode Morphological Identification

Fifty-six soil samples were gathered primarily from the rhizosphere of maritimus pine (*Pinus pinaster* Ait.) forests and one single sample from green heather (*Erica scoparia* L.) in three mountains (including Bermeja-Crestellina, Nieves and Tejeda-Almijara Mountains, located at western, central and eastern part of Malaga province) belonging to five municipalities (Casares, Tolox, Igualeja, Canillas de Albaida, and Carratraca) in the Malaga province, Southern Spain (Table 1). Samples were taken using a shovel and considering the upper 5–40 cm depth of soil. Nematodes were analyzed from a 500-cm^3^ subsample of soil by centrifugal flotation [50].

Morphological and morphometrical analyses included a total of 137 specimens, comprising 124 females, 1 male and 12 juveniles. Individuals for light microscopy (LM) analysis were killed and fixed in an aqueous solution of 4% formaldehyde + 1% glycerol, dehydrated using alcohol-saturated chamber and processed to pure glycerine using Seinhorst’s method [51] according to De Grisse [52]. The life-stage of the juveniles for the undescribed species was identified considering the body length and the grade of progress of genital cells [22]. Light micrographs were taken using fresh nematodes and measurements of each nematode population, including significant diagnostic characteristics (i.e., de Man indices, body length, stylet length, lip region, tail shape) [53], were completed by means of a Leica DM6 compound microscope with a Leica DFC7000 T digital camera (Wetzlar, Germany) and comprising fixed and mounted nematodes in glycerin. Nematodes were identified at specific levels applying an integrative taxonomy merging morphological techniques (including the recent web-assisted polytomous key of Palomares-Rius et al. [8]) and molecular analyses to achieve an efficient and accurate identification [3,4,5]. Within each nematode population, significant diagnostic traits were evaluated, comprising body length, stylet length, a ratio (body length/maximum body diameter), b ratio (body length/total pharyngeal length), c ratio (body length/tail length), c’ ratio (tail length/body width at anus level), V ratio ((distance from anterior end to vulva level/body length) × 100), and o ratio ((distance from stylet base to dorsal pharyngeal orifice/stylet length) × 100) [3,4,5], and the sequencing of specific molecular markers (listed below) corroborated the distinctiveness of the nematode species for individual populations.

Nematode populations of *Paratylenchus* species previously described and molecularly analyzed in this study for the first time were recommended as accepted and referral populations until topotype material for separate species becomes available and molecularly characterized. Voucher individuals of these defined species have been maintained in the nematode collection of Institute for Sustainable Agriculture, IAS-CSIC, Córdoba, Spain.

### 4.2. DNA Extraction, PCR and Sequencing

DNA extraction was always based on single nematode specimens as defined by Palomares-Rius et al. [54], and more decisive, for all the 27 considered populations, all the three molecular markers of each *Paratylenchus* population are coming from the same single DNA extracted nematode in individually PCR tube without any exemption. Furthermore, assignation of male and juvenile stages to one species always was proven by single DNA extraction of these individuals. Additionally, for avoiding mistakes, in the case of mixed *Paratylenchus* populations within the same soil sample (being common in this study), single nematodes were provisionally deposited in a drop of 1 M NaCl containing glass beads (to avoid nematode crushing/damaging specimens) to ensure specimens were coincident with the unidentified population. This saline solution did not affect the morphology of nematodes.

The D2 and D3 expansion domains of the 28S rRNA were amplified using the D2A (5′-ACAAGTACCGTGAGGGAAAGTTG-3′) and D3B (5′-TCGGAAGGAACCAGCTACTA-3′) primers [55]. The Internal Transcribed Spacer region (ITS) was amplified using forward primer TW81 (5′- GTTTCCGTAGGTGAACCTGC -3′) and reverse primer AB28 (5′-ATATGCTTAAGTTCAGCGGGT -3′) [56]. The COI gene was amplified using the primers JB3 (5′-TTTTTTGGGCATCCTGAGGTTTAT-3′) and JB5 (5′-AGCACCTAAACTTAAAACATAATGAAAATG-3′) [57]. The PCR cycling conditions for all three molecular markers were as described in Clavero-Camacho et al. [5], De Ley et al. [55], Subbotin et al. [56] and Bowles et al. [57]. In all PCR reactions, we used 5× HOT FIREpol Blend Master Mix (Solis Biodyne, Tartu, Estonia). ExoSAP-IT (Affimetrix, USB products, Kandel, Germany) was used to purify the PCR products and used for direct sequencing in both directions with the corresponding primers. The subsequent products were run in a DNA multicapillary sequencer (Model 3130XL Genetic Analyzer; Applied Biosystems, Foster City, CA, USA), using the BigDye Terminator Sequencing Kit v.3.1 (Applied Bio-systems) at the Stab Vida sequencing facility (Caparica, Portugal). The sequence chromatograms of the 3 markers (D2-D3 expansion segments of 28S rRNA, ITS rRNA, and COI) were analyzed using DNASTAR LASERGENE SeqMan v. 7.1.0. Basic local alignment search tool (BLAST) at the National Center for Biotechnology Information (NCBI) was used to confirm the species identity of the DNA sequences obtained in this study [58]. The newly obtained sequences were sent to the GenBank database under accession numbers shown on the phylogenetic trees and in Table 1.

### 4.3. Phylogenetic Analyses

In this study, D2-D3 expansion segments of 28S rRNA, ITS rRNA, and COI mtDNA fragments of the 27 *Paratylenchus* populations were sequenced. Obtained sequences and other from species of *Paratylenchus* from NCBI were employed for phylogenetic analyses. For each dataset, the outgroup taxa selection was constructed according to previously published phylogenies and considering the molecular diversity of each dataset [3,5,29,42,59]. FFT-NS-2 algorithm of MAFFT V.7.450 [60] was used for multiple sequence alignments of the different genes. BioEdit program V. 7.2.5 [61] was used for sequence alignments visualization. Alignments were manually edited and trimmed of the poorly aligned positions, using a light filtering strategy (up to 20% of alignment positions), which has little impact on tree accuracy and may save some computation time, as suggested by Tan et al. [62]. Methods for automated filtering of multiple sequence alignments frequently worsen single-gene phylogenetic inference [62]. Bayesian inference (BI) applying MrBayes 3.1.2 [63] was used for phylogenetic analyses of the sequence datasets. JModelTest V.2.1.7 [64] with the Akaike information criterion (AIC) was used to get the best-fit model of DNA evolution. The best-fit model, the base frequency, the proportion of invariable sites, and the gamma distribution shape parameters and substitution rates in the AIC were then used in MrBayes for the phylogenetic analyses. The general time-reversible model with invariable sites and a gamma-shaped distribution (GTR + I + G) for the D2-D3 segments of 28S rRNA, the partial ITS rRNA, and COI gene, were run with four chains for 4, 4, and 10 × 10^6^ generations, respectively. A joint analysis of the two ribosomal genes was not performed due to some sequences not being accessible for all species. The Markov chains were sampled at intervals of 100 generations. For each analysis, two runs were conducted. After discarding burn-in samples of 30% and evaluating convergence, the remaining samples were retained for more in-depth analyses. The topologies were used to generate a 50% majority-rule consensus tree. On each appropriate clade, posterior probabilities (PP) were given. FigTree software version v.1.42 [65] was used for visualizing trees from all analyses.

## 5. Conclusions

This research proves and emphasizes the importance of using integrative taxonomy for the accurate identification of *Paratylenchus* species in complex scenarios such as wild environments. Our results also establish the presence of further cryptic biodiversity within the *P. straeleni*-complex species, augmenting and increasing the diversity of these plant-parasitic nematodes in Spain. This study delivers ribosomal and mitochondrial markers for accurate and unambiguous diagnosis of *P. straeleni*-complex and advises that other reports of *P. straeleni* in Spain and all over the world need to be confirmed with molecular markers. In addition, these data also indicate that species diversity in natural environments may be higher than that in cultivated areas, since two new *Paratylenchus* species to science and six first reports were detected with respect to previous studies, two of them new species for science (*P. paraaonli* sp. nov., *P. plesiostraeleni* sp. nov.), and six species are considered as first reports for Spain in this study (*viz*. *P. canchicus*, *P. nainianus*, *P. neonanus*, *P. salubris*, *Paratylenchus* sp. 2 SAS, and *P. wuae*). Then, our data endorse the anticipated hypothesis that until now we have only elucidated to barely a minimal part of the biodiversity within *Paratylenchus* described in Spain in wild habitats and possibly worldwide.

## Figures and Tables

**Table 1:** *Paratylenchus* species identified in the rhizosphere of maritimus pine (*Pinus pinaster* Ait.) and green heather (*Erica scoparia* L.) from three mountains of the Malaga province, southern Spain.

Paratylenchus Species	Sample Code †	Locality, Province	D2-D3	ITS	COI
***Paratylenchus paraaonli* sp. nov.**	WPPp3 *	Casares, Málaga	ON873196-ON873199	-	ON873944, ON873945
***Paratylenchus paraaonli* sp. nov. (type population)**	WPPp4 *	Casares, Málaga	ON873200-ON873203	ON873174-ON873178	ON873946-ON873952
***Paratylenchus plesiostraeleni* sp. nov. (type population)**	CMPp4 *	Tolox, Málaga	ON873204-ON873207	ON873179-ON873182	ON873954-ON873957
***Paratylenchus plesiostraeleni* sp. nov.**	WPPp4 *	Casares, Málaga	ON873208	ON873183	ON873953
***Paratylenchus plesiostraeleni* sp. nov.**	EMPp6 *	Canillas de Albaida, Málaga	ON873209-ON873211	ON873184, ON873185	ON873958-ON873963
*Paratylenchus canchicus* Mohilal and Dhanachand, 2004	WMPp1 *	Casares, Málaga	ON873212, ON873213	-	ON873964, ON873965
*Paratylenchus caravaquenus* Clavero-Camacho et al., 2021	ECPp2 *	Canillas de Albaida, Málaga	ON873214	-	-
*P. caravaquenus*	EMPp3 *	Canillas de Albaida, Málaga	ON873215	-	-
*Paratylenchus microdorus* Andrássy, 1959	WMPp1 *	Casares, Málaga	ON873231, ON873232		
*P. microdorus*	WPPp1 *	Casares, Málaga	ON873230	ON873190	ON873979
*Paratylenchus nanus* Cobb, 1923	EPPp4 *	Carratraca, Málaga	ON873216	-	-
*Paratylenchus nainianus* Edward & Misra, 1963	BRZE1 **	Casares, Málaga	ON873217-ON873220	ON873186-ON873189	ON873966-ON873969
*Paratylenchus nainianus*	WMPp3 *	Casares, Málaga	ON873221, ON873222		ON873970-ON873975
*Paratylenchus neoamblycephalus* Geraert, 1965	EPPp5 *	Carratraca, Málaga	ON873223, ON873224	-	-
*P. neoamblycephalus*	CPPp4 *	Igualeja, Málaga	ON873225	-	-
*Paratylenchus neonanus* Mathur et al., 1967	WPPp4 *	Casares, Málaga	ON873226-ON873229	-	ON873976-ON873978
*Paratylenchus salubris* Raski, 1975	WPPp3 *	Casares, Málaga	ON873233-ON873235	-	ON873980-ON873983
*Paratylenchus sheri* (Raski, 1973) Siddiqi, 1986	EMPp6 *	Canillas de Albaida, Málaga	ON873236	-	-
*P. sheri*	EPPp4 *	Carratraca, Málaga	ON873237	-	-
*P. sheri*	CPPp1 *	Tolox, Málaga	ON873238	-	-
*P. sheri*	CPPp5 *	Tolox, Málaga	ON873239	-	-
*P. sheri*	CPPp2 *	Tolox, Málaga	ON873240		
*P. sheri*	WCPp1 *	Casares, Málaga	ON873241	-	-
*Paratylenchus* sp. 2 SAS	CPPp5 *	Tolox, Málaga	ON873242-ON873245	ON873191-ON873193	ON873984-ON873987
*Paratylenchus variabilis* Raski, 1975	EMPp1 *	Canillas de Albaida, Málaga	ON873246	-	-
*Paratylenchus wuae* Yu et al., 2016	WPPp3 *	Casares, Málaga	ON873247-ON873249	-	ON873988-ON873990
*Paratylenchus wuae*	EPPp4 *	Carratraca, Málaga	ON873250, ON873251	ON873194, ON873195	ON873991-ON873994

(†) Sample codes First capital letter: W = western area of Malaga province, Bermeja-Crestellina Mountain; C = central area of Malaga province, Nieves Mountain; E = eastern area of Malaga province, Tejeda-Almijara. * maritimus pine (*Pinus pinaster* Ait.). ** green heather (*Erica scoparia* L.). (-) Not obtained or not performed.

## Data Availability

The datasets generated during and/or analyzed during the current study are available NCBI and from the corresponding author on reasonable request.
